# Paper and Flexible Substrates as Materials for Biosensing Platforms to Detect Multiple Biotargets

**DOI:** 10.1038/srep08719

**Published:** 2015-03-06

**Authors:** Hadi Shafiee, Waseem Asghar, Fatih Inci, Mehmet Yuksekkaya, Muntasir Jahangir, Michael H. Zhang, Naside Gozde Durmus, Umut Atakan Gurkan, Daniel R. Kuritzkes, Utkan Demirci

**Affiliations:** 1 Demirci Bio-Acoustic-MEMS in Medicine (BAMM) Laboratory, Division of Biomedical Engineering, Division of Renal Medicine, Department of Medicine, Brigham and Women's Hospital, Harvard Medical School, Boston, MA, USA; 2 Demirci Bio-Acoustic-MEMS in Medicine (BAMM) Laboratory, Stanford University School of Medicine, Canary Center at Stanford for Cancer Early Detection, Palo Alto, California, USA; 3 Department of Biochemistry, Stanford School of Medicine, Stanford, California, USA; 4 Stanford Genome Technology Center, Stanford University, Palo Alto, California, USA; 5 Division of Infectious Diseases, Brigham and Women's Hospital, Harvard Medical School, MA, USA

## Abstract

The need for sensitive, robust, portable, and inexpensive biosensing platforms is of significant interest in clinical applications for disease diagnosis and treatment monitoring at the point-of-care (POC) settings. Rapid, accurate POC diagnostic assays play a crucial role in developing countries, where there are limited laboratory infrastructure, trained personnel, and financial support. However, current diagnostic assays commonly require long assay time, sophisticated infrastructure and expensive reagents that are not compatible with resource-constrained settings. Although paper and flexible material-based platform technologies provide alternative approaches to develop POC diagnostic assays for broad applications in medicine, they have technical challenges integrating to different detection modalities. Here, we address the limited capability of current paper and flexible material-based platforms by integrating cellulose paper and flexible polyester films as diagnostic biosensing materials with various detection modalities through the development and validation of new widely applicable electrical and optical sensing mechanisms using antibodies and peptides. By incorporating these different detection modalities, we present selective and accurate capture and detection of multiple biotargets including viruses (Human Immunodeficieny Virus-1), bacteria (*Escherichia coli and Staphylococcus aureus)*, and cells (CD4^+^ T lymphocytes) from fingerprick volume equivalent of multiple biological specimens such as whole blood, plasma, and peritoneal dialysis effluent with clinically relevant detection and sensitivity.

Rapid diagnosis has broad applications in multiple disciplines including clinical medicine, immunoassays, food safety, environmental monitoring, and veterinary medicine^1^,^2^,^3^,^4^,^5^. Among these applications, development of easy-to-use, rapid, and inexpensive point-of-care (POC) diagnostic assays, particularly in resource-constrained settings, remains an urgent need. POC diagnostics facilitate timely opportunities for detection and control of disease, treatment monitoring and counseling in both developed and developing countries to solve real world healthcare problems^6^,^7^. Paper and flexible material-based platform technologies have created an exciting avenue in the area of POC diagnostics. These technologies have also potential to shift the paradigm in developing diagnostic assays, particularly for the developing world, as they are thin, light, flexible, inexpensive, and materials are readily available around the world. These platforms are easy-to-fabricate, mass-producible, easy-to-use, and disposable^1^,^8^. Paper-based platforms can also absorb fluid samples through capillary effect, and be incinerated for convenient and safe disposal^8^. Therefore, these platforms provide attractive strategies for developing affordable tools with broad applications such as drug development, water and environment quality monitoring, and infectious diseases diagnosis in resource-constrained settings^8^,^9^. Current paper and flexible material-based platforms utilizing colorimetric^10^, fluorometric^11^, and electrochemical approaches^12^ require complex labeling steps to amplify the signal. Further, these platforms need expensive infrastructure and skilled personnel for their fabrication and operation, which are significant challenges for their deployment into the POC settings. Although there has been a considerable number of studies on paper and flexible material-based biosensors, there is a need for studies demonstrating (i) their applicability to different detection modalities such as electrical and optical sensing, and (ii) their detection capability for various biotargets from a variety of clinical sample types including whole blood, serum, saline, dialysis effluent or lysates with high sensitivity and specificity. By employing three different paper and flexible material-based platforms incorporated with electrical and optical sensing modalities, we address these major challenges with specific examples for their broad use as diagnostic device materials.

In this manuscript, we demonstrate different paper and flexible material-based diagnostic platforms with biosensing applications to detect various bioagents in whole blood, serum and peritoneal fluid. These distinct platforms have been integrated with sensing modalities including impedance spectroscopy of biotargets, nanoparticle aggregation using a mobile phone-based detection system, and lensless shadow imaging technology that do not require any signal amplification. As clinical disease models, we present applications of paper and flexible material-based platforms focusing on (i) detection of Human Immunodeficiency Virus-1 (HIV-1) by capturing and detecting multiple HIV-1 subtypes, (ii) CD4^+^ T lymphocytes, and (iii) detection of bacterial infections such as *Escherichia coli* (*E. coli*) and *Staphylococcus aureus* (*S. aureus*) in various clinical matrices with high sensitivity and specificity. Thus, we demonstrate that paper and flexible material-based platforms can be widely applied to a variety of settings including medical diagnostic and biology laboratories. The combination of these sensing mechanisms on paper and flexible materials offers a promising opportunity to create diagnostic assays that meet the requirements of POC disease detection and treatment monitoring.

## Results

### HIV detection on a flexible polyester film-based electrical sensing platform

**Figure 1:**
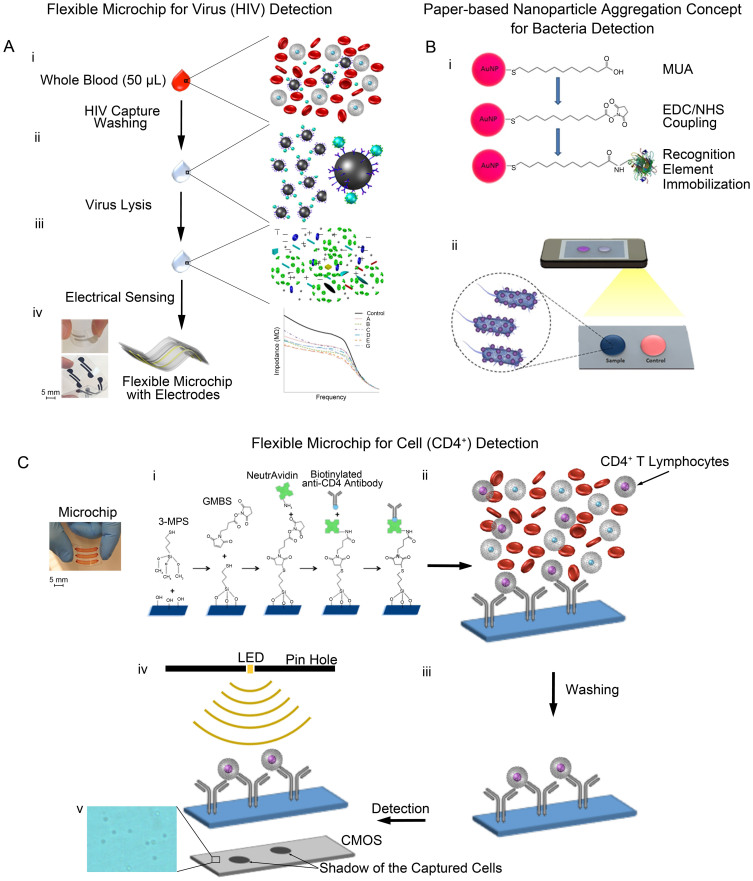
Schematic of seamlessly integrated paper and flexible substrate-based platforms with broadly applicable electrical and optical sensing modalities. (A) Flexible electrodes on polyester film-based electrical sensing platform for HIV detection. (i) Viruses are captured using streptavidin-coated magnetic beads conjugated with biotinylated polyclonal anti-gp120 antibodies in clinically relevant samples such as whole blood (ii) Sample is washed to remove electrically conductive solution. (iii) Captured viruses are lysed using 1% Triton X-100, releasing to release ions and biomolecules of the captured viruses. These biomolecules change the electrical conductivity of the solution. (iv) The impedance magnitude of the viral lysate samples decreases compared with that of control samples. Figure 1A is drawn by H.S. (B) Nanoparticle aggregation concept for bacteria detection on a cellulose paper and its incorporation with mobile phone camera. (i) Gold nanoparticles (AuNPs) are modified with 11-Mercaptoundeconoic acid (MUA), N-Ethyl-N'-(3-dimethylaminopropyl) carbodiimide hydrochloride (EDC), and N-hydroxysuccinimide (NHS) to form coupling groups on nanoparticle surface. AuNPs are further functionalized with specific recognition elements for bacteria pathogens. (ii) Bacteria-spiked samples are added to the modified nanoparticle solution. Bacteria samples cause nanoparticle aggregation and change the color of solution as detected with a mobile phone. Figure 1B is drawn by F.I. (C) Lensless imaging detection and counting of CD4^+^ T lymphocytes on polyester film-based platform with microchannels. (i) Surface chemistry on the platform are designed to immobilize biotinylated anti-CD4 antibodies using NeutrAvidin on chemically activated surface including N-g-Maleimidobutyryloxy succinimide ester (GMBS) and 3-Mercaptopropyl trimethoxysilane (3-MPS) modifications. (ii) CD4^+^ T lymphocytes are selectively captured from a whole blood sample on the surface of platform. (iii) Non-captured cells are washed away from the microchannels of platform. (iv) Captured CD4^+^ T cells are then detected and quantified using a lensless shadow imaging technology. (v) Shadows of captured CD4^+^ T cells on the substrate. Figure 1C is drawn and designed by H.S., W.A. and M.H.Z.

**Figure 2:**
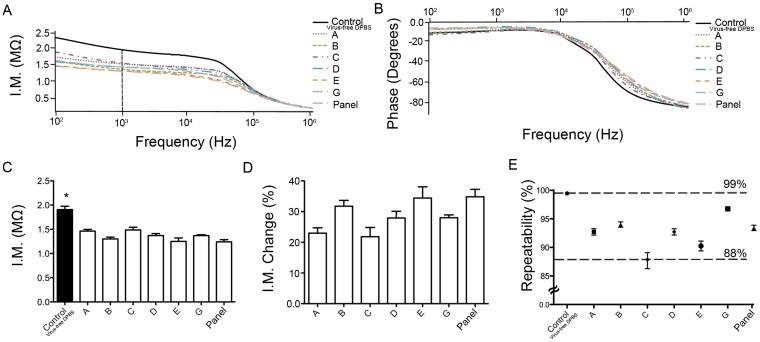
Impedance spectroscopy results for HIV-spiked DPBS samples. Impedance magnitude (I.M.) (A) and phase spectra (B) of lysed HIV-1 subtypes A, B, C, D, E, G, and panel for frequencies between 100 Hz and 1 MHz measured on the flexible polyester film-based electrical sensing devices. (C) Impedance magnitude of HIV-1 lysate samples (HIV subtypes A, B, C, D, E, G, and panel) at 1,000 Hz and 1 V, where there was maximum impedance magnitude shift. (D) Normalized impedance magnitude change of HIV-1 lysate samples with respect to control samples. (E) Repeatability of the measured impedance magnitude for HIV subtypes A, B, C, D, E, G, and panel. The system demonstrated a repeatability between 88% and 99%. The control samples were virus-free DPBS samples. Statistical assessment on the results was performed using ANOVA with *Tukey's posthoc* test for multiple comparisons. Statistical significance threshold was set at 0.05 (p < 0.05). Error bars represent standard deviation of the mean (n = 3). Asterisk indicates statistically significant impedance magnitude change compared to all other groups. The viral loads of these HIV-1 subtypes were 1.74 × 10^8^, 1.2 × 10^8^, 1.17 × 10^8^, 2.9 × 10^8^, 8.39 × 10^8^, 6.53 × 10^8^, and 1.49 × 10^9^ copies/mL for HIV subtypes A, B, C, D, E, G, and panel, respectively.

A flexible polyester film with two rail silver electrodes in microfluidic channels was employed as an electrical sensing platform to detect viruses (HIV-1) through viral lysate impedance spectroscopy (Figs. 1A, S1, and S2). Here, biological samples spiked with HIV-1 were used as a clinical disease model for HIV-1 diagnosis. HIV-1 spiked in samples were first captured and isolated off-chip using magnetic beads conjugated with biotinylated polyclonal anti-gp120 antibodies. To detect captured HIV-1, we evaluated the impedance magnitude and phase spectra of viral lysate samples for multiple HIV-1 subtypes including A, B, C, D, E, G, and panel over a range of frequencies between 100 Hz and 1 MHz (Figs. 2A and 2B). Our preliminary experiments were performed using HIV-spiked into Dulbecco's phosphate buffered saline (DPBS) samples. Captured viruses were then detected through viral lysate impedance spectroscopy on the platform. Viral lysis step increases the electrical conductivity and decreases the bulk impedance magnitude of the solution. An equivalent electronic circuit model was developed to calculate the impedance magnitude of the viral lysate samples (Fig. S3) as earlier developed^13^. The average impedance magnitude spectra of the viral lysate samples for all HIV-1 subtypes were below the average impedance magnitude spectrum of the control samples for frequencies between 100 Hz and 100 kHz (Fig. 2A). Control samples were prepared by mixing HIV-free DPBS and conjugated magnetic beads. The impedance magnitude of the viral lysate samples for frequencies above 100 kHz was same as the impedance magnitude of the control samples (Fig. 2A). We observed that the maximum impedance magnitude shift for all HIV-1 subtypes with respect to control samples occurred at 1,000 Hz (Fig. 2A). Therefore, we chose this frequency to further statistically compare the impedance magnitude results. Fig. 2C shows the impedance magnitude of the viral lysate samples as well as control sample at 1,000 Hz. The impedance magnitude of the viral lysate samples was significantly lower than the impedance magnitude of the control samples (n = 3, p < 0.05), indicating that the captured and lysed viruses changed the electrical conductivity of the solution, thus can be detected using impedance spectroscopy (Fig. 2C). We also observed no significant difference between the impedance magnitudes of multiple HIV-1 subtypes (Figs. 2C and 2D, n = 3, p > 0.05), which indicated that this method of virus sensing was able to detect multiple HIV subtypes. The impedance magnitude of the viral lysate samples were also normalized with respect to the impedance magnitude of control samples at 1,000 Hz as shown in Fig. 2D. These results showed an impedance magnitude change of 20% to 30% compared with the control samples. The repeatability of the impedance magnitude measurements was observed to be between 88% and 99% (Fig. 2E and Supplementary Information). We evaluated the virus capture on magnetic beads conjugated with antibodies using green fluorescent protein (GFP)-tagged HIV-1 (Fig. S4). We also investigated the effect of time on the impedance magnitude of the viral lysate samples, and we did not observe a significant difference in the impedance magnitude of the samples during the time of performing experiments (n = 3, p > 0.05) (Fig. S5).

**Figure 3:**
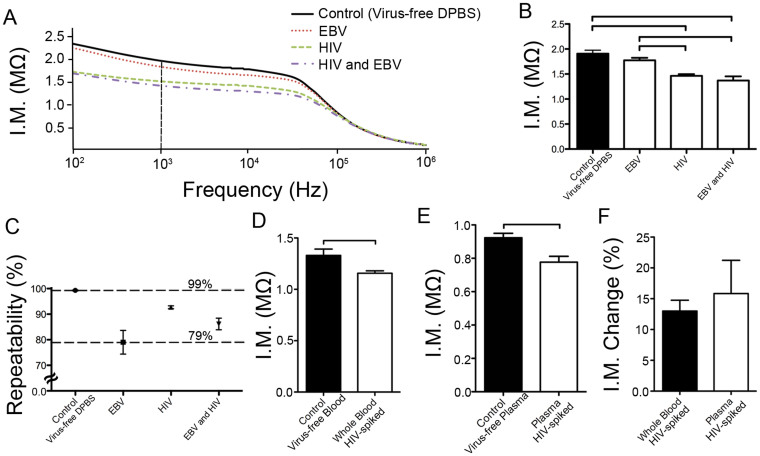
Evaluation of specificity characteristics for the flexible material (*i.e.*, polyester film)-based electrical sensing platform. EBV- and HIV-spiked DPBS samples were used in the experiments. Control samples were virus-free DPBS. Average impedance magnitude (A) for lysed HIV-1 subtype A, EBV, and mixture of HIV subtype A and EBV for frequencies between 100 Hz and 1 MHz. (B) Impedance magnitude of the lysed HIV-1 subtype A, EBV, and mixture of HIV subtype A and EBV samples at 1,000 Hz and 1 V. (C) Repeatability of the impedance magnitude shift for HIV-1 subtype A, EBV, and mixture of HIV and EBV samples. The system demonstrated repeatability between 79% and 99%. Average impedance magnitude of control and lysed HIV-1 subtype C spiked in whole blood (D) and plasma (E) at 1,000 Hz and 1 V. The control samples were virus-free blood and virus-free plasma for experimental results reported in (D) and (E), respectively. Error bars represent standard error of the mean (n = 3). (F) Normalized impedance magnitude change of HIV-1 lysate samples with respect to control samples at 1,000 Hz and 1 V in spiked whole blood and plasma samples. Statistical assessment on the results was performed using ANOVA with *Tukey's posthoc* test for multiple comparisons. Statistical significance threshold was set at 0.05 (p < 0.05). Error bars represent standard error of the mean (n = 3). Brackets connecting individual groups indicate statistically significant impedance magnitude difference (p < 0.05). The viral loads of the HIV-1 subtype A and C used here were 1.74 × 10^8^, 1.17 × 10^8^ copies/mL, respectively.

To evaluate the specificity of this platform for HIV-1 detection, we used Epstein-Barr virus (EBV). The impedance magnitude spectra of HIV-1, EBV, and the mixture of HIV-1 and EBV samples were measured and compared with control samples (Fig. 3A). Controls were virus-free DPBS samples. The impedance magnitude spectra of the HIV-1 and the mixture samples were lower compared to control and EBV samples (Fig. 3A). Further, the mean impedance magnitude spectrum of EBV samples was not significantly lower than control samples (Fig. 3A). The impedance magnitude of the samples was then analyzed at 1,000 Hz. The impedance magnitude of HIV-1 and the mixture of HIV-1 and EBV samples were significantly different compared to control samples at 1,000 Hz (Fig. 3B, n = 3, p < 0.05). The impedance magnitude of HIV-1 samples was not statistically different than the mixture of HIV-1 and EBV at 1,000 Hz (Fig. 3B, n = 3, p > 0.05). We also observed that the repeatability of these impedance magnitude measurements were between 79% and 99% (Fig. 3C).

To further evaluate the sensing ability of the platforms in complex biological samples, HIV samples were selectively isolated and detected from whole blood and plasma. Spiked viruses into whole blood and plasma samples were captured and isolated using biotinylated polyclonal anti-gp120 antibodies, washed 4 times with 20% glycerol solution, and lysed for impedance spectroscopy detection (Fig. S6). The impedance magnitude spectra of the viral lysate samples are shown in Figs. S6A and S6B. The impedance magnitude of viral lysate samples were significantly lower compared to the impedance magnitude of control samples (whole blood and plasma without viruses) at 1,000 Hz and 1 V (Figs. 3D and 3E, n = 3, p < 0.05). The impedance magnitude shift in whole blood and plasma samples was found to be 13 ± 2% and 16 ± 5%, respectively (Fig. 3F). No significant difference was observed between the impedance magnitude shift in whole blood and plasma samples, indicating that the viruses were detected both in plasma and whole blood (Fig. 3F, n = 3, p > 0.05). Further, using higher sample volumes up to 5 mL increased the sensitivity of the platform, where the impedance magnitude of the viral lysate samples was significantly lower than control samples (virus-free DPBS samples) (Fig. S7, n = 3, p < 0.05). We were able to detect viruses using this polyester film-based electrical sensing platform in HIV-spiked DPBS samples with viral loads on the order of 10^6^ copies/mL (Fig. S7).

Electrical sensing is a powerful modality to develop portable, rapid, and sensitive diagnostic tools^2^,^3^,^13^,^14^,^15^. By employing polyester film as a flexible material, this label-free platform can potentially be used for the capture and detection of multiple pathogens with well-known biomarkers using impedance spectroscopy of their lysate. Here, we particularly present the ability of this platform to detect HIV-1 at the viral concentrations matching acute HIV-1 infection stage, where there is maximum virus reproduction (10^6^–10^8^ copies/mL) and minimum antibody concentration^16^. HIV-infected individuals can transmit the infection substantially during acute stage of HIV infection^17^. Thus, acute HIV-1 detection is crucial, where current rapid tests such as OraQuick or Determine HIV-1/2 Ag/Ab Combo rapid test cannot detect HIV infection due to low concentration of antibodies before the seroconversion, and poor sensitivity to detect acute HIV in field studies^16^,^18^,^19^. These statistics clearly highlight the urgent and unmet clinical need for a POC viral load test for acute HIV detection^20^. Here, we have developed an inexpensive (less than $2 in material cost) (Table S1), and disposable electrical sensing platform on a flexible material (*i.e.*, polyester film) to selectively capture and detect viruses (HIV-1) in clinical samples such as whole blood and plasma. However, this platform requires off-chip sample preparation including virus capture using antibodies, washing, and virus lysis. By employing further developments in sample processing steps, this platform technology can be potentially automated for POC applications.

### Bacteria detection and quantification on a cellulose paper-based nanoparticle aggregation substrate and image acquisition with a mobile phone

An ultra-sensitive biosensing platform was developed to detect pathogens through nanoparticle aggregation on a cellulose paper (Fig. 1B). The nanoparticle aggregation strategy was adapted as a sensing mechanism for detection of *E. coli and S. aureus* as model bacterial pathogens. In this platform, gold nanoparticles were first modified with specific recognition elements to capture pathogens in the solution. We subsequently transferred the modified nanoparticle solution to a disposable paper that allowed distribution of aggregated nanoparticles by utilizing capillary effect on its native structure. thus obtaining uniform distribution for quantitative analysis. To evaluate the chemical modifications on nanoparticle surface, 11-Mercaptoundeconoic acid (MUA), N-Ethyl-N'-(3-dimethylaminopropyl) carbodiimide hydrochloride (EDC)/N-hydroxysuccinimide (NHS) coupling agents, and Lipopolysaccharide Binding Protein (LBP) for recognition of *E. coli* were used to modify gold nanoparticles. The maximum extinction wavelength peaks of nanoparticle colloids resulted in red-shifts after each surface modification (Fig. S8A). The conjugated nanoparticles were mixed with *E. coli*-spiked solutions, and the nanoparticles then aggregated due to *E. coli*-nanoparticle interactions (Figs. S8B and S9). Collectively, the results in Fig. S8 demonstrated that the final morphology, structure, and status of the nanoparticle aggregation was acutely triggered by the presence of *E. coli* in the solution, which caused a visible change in the color from red to blue.

**Figure 4:**
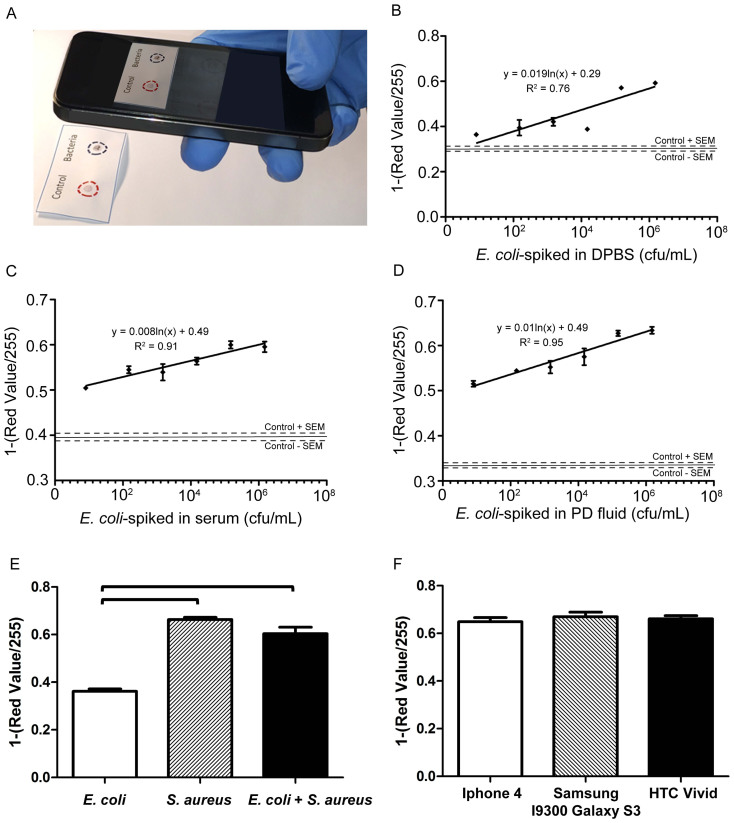
Cellulose paper-based nanoparticle aggregation for the detection of bacteria with a mobile phone system. Bacteria-spiked samples were first added into the modified gold nanoparticle solution, and then, transferred to a cellulose paper for distribution of nanoparticles due to capillary effect. (A) The images of sample spots were taken using a mobile phone camera for image acquisition, and analyzed using an image processing MATLAB code in the computer. Red (R) pixel intensity value was used in the data analysis. (B) *E. coli*-spiked into DPBS samples ranging from 8 to 1.5 × 10^6^ CFUs/mL were evaluated. The limit of detection (LOD) was observed to be 8 CFUs/mL (n = 5). (C) *E. coli*-spiked into serum samples ranging from 8 to 1.5 × 10^6^ CFUs/mL were evaluated. The LOD was observed as 8 CFUs/mL (n = 5). (D) *E. coli*-spiked in PD fluid samples ranging from 8 to 1.5 × 10^6^ CFUs/mL were evaluated. The LOD was observed as 8 CFUs/mL (n = 5). *E. coli*-free solutions were used as control samples, and the corresponding signals were represented in line and dashes in the plots to show the mean and standard error of mean, respectively (n = 5). (E) Three sets of experiments were designed for multiplexing and specificity experiments. *S. aureus* (10^5^ CFUs/mL) were applied into anti-LTA antibody-modified gold nanoparticle solution for multiplexing. *E. coli* (10^5^ CFUs/mL) were sampled into anti-LTA antibody-modified gold nanoparticle solution for specificity. A mixed bacteria solution was examined with anti-LTA antibody-modified gold nanoparticle solution for nonspecific interactions. Statistical analysis demonstrated that *S. aureus* and mixture experiments were statistically different than *E. coli* samples (n = 5, p < 0.05), and there was no significant difference observed between *S. aureus* and mixture samples (n = 5, p > 0.05). (F) Evaluation of image code with other mobile phone cameras. *S. aureus* experiments were further assessed with iPhone 4, Samsung i9300 Galaxy S3 and HTC Vivid. There was no statistical difference between various mobile phone cameras (n = 5, p > 0.05). Statistical analysis was described in Supplementary Information. Brackets connecting individual groups indicate statistically significant difference. Error bars represent standard error of the mean.

To this end, the platform was validated by spiking E. coli into three distinct media: DPBS, whole blood serum, and peritoneal dialysis (PD) fluid (Fig. 4). These three media without E. coli were used as controls. The blue color was obtained in *E. coli*-spiked samples, and it was also distinguished from control samples (red color) (Fig. 4A). This color change in nanoparticles is associated with the interference of biomolecular interactions between the intermolecular interactions. Control experiments (*E. coli*-free samples in DPBS, whole blood serum, and PD fluid) resulted in red-colored colloids of nanoparticles since there is no recognition site for LBP protein in the solution. Therefore, these experiments showed that blue color formation was not due to non-specific interactions, and it was observed in the presence of target bacteria (*E. coli*). For quantitative analysis, the solution containing modified nanoparticles and bacteria was also sampled and dried on a paper within 10 minutes, and a mobile phone camera (Sony Ericson i790) was then employed as an image acquisition step for nanoparticle aggregate spots (Fig. 1B). Further, the ability to detect *E. coli* with a naked eye was confirmed by measuring the intensity with a customized imaging analysis tool on the computer, which provided color intensities of images using a customized MATLAB code in terms of red (R), green (G), and blue (B) pixel values as previously presented^21^. Briefly, the code was used (i) to obtain RGB pixel values and (ii) to calculate color intensity for each image. In these experiments, R value allowed a broader color intensity range, and thus, we continued to employ R values for measurements. The area of interest was manually selected in the middle of nanoparticle aggregate spots with ~0.25 cm^2^ on the cellulose paper surface. Further, as we demonstrated previously^21^,^22^, this code can also be adapted to a mobile phone application for automatic region selection (given a specific region is marked on the paper) and analysis.

To evaluate the limit of detection, control and *E. coli* samples were analyzed using statistical methods, and the lowest detectable concentration was observed to be 8 colony forming units (CFUs)/mL for all samples (Fig. 4B–D). These results demonstrated the employment of the nanoparticle aggregation approach-operated on a cellulose paper for the detection and identification of pathogens at low concentrations. For multiplexing and specificity experiments, we designed three sets of experiments: (i) the sampling of *S. aureus* (10^5^ CFUs/mL) into anti-LTA antibody-modified gold nanoparticle solution, (ii) the sampling of *E. coli* (10^5^ CFUs/mL) into anti-LTA antibody-modified gold nanoparticle solution, and (iii) the sampling of mixed bacteria (*S. aureus* (10^5^ CFUs/mL) and *E. coli* (10^5^ CFUs/mL)) into anti-LTA antibody-modified gold nanoparticle solution (Fig. 4E). From multiplexing perspective, *S. aureus*-spiked samples presented statistically different results than that of *E. coli*-spiked samples (n = 5, p < 0.05) (Fig. 4E). Further, we observed a minimal cross-reactivity (non-specific binding) against *E. coli* that is statistically smaller than the data in mixture samples (n = 5, p < 0.05) (Fig. 4E). At the third set of experiments, we evaluated the specificity performance of the platform with mixed samples. Statistical analysis demonstrated that there was no significant difference between the results obtained from *S. aureus* and mixture samples (n = 5, p > 0.05) (Fig. 4E). Since there is no notable interaction between anti-LTA antibody-modified gold nanoparticles and *E. coli*, the color intensity originated due to *S. aureus* confirmed selective capture and detection of *S. aureus* from the mixed samples (Fig. 4E). In addition, to assess the MATLAB code performance for analysis, *S. aureus* experiments were further evaluated with other mobile phones such as iPhone 4, Samsung i9300 Galaxy S3 and HTC Vivid, which have different cameras for the image acquisition (Fig. 4F). Without any modification on the code, we did not observe any statistical difference between the results obtained from various mobile phone cameras (n = 5, p > 0.05). Overall, we demonstrated a specific and multiplexed platform for the detection of different bacterial pathogens using an unconventional nanoparticle aggregation approach incorporated with cellulose paper.

From a diagnostics perspective, colorimetric ELISA-based detection assays are currently employed as a conventional laboratory method, where the corresponding detection signal is formed by the conversion of enzyme substrate into a colored product, and the signal intensity is measured using a spectroscopic plate reader^21^. Although the color changes can be distinguished by a naked eye, these assays require multiple operational steps and expensive infrastructure. In our assay, the formation of colored solutions is employed to quantitatively detect target pathogens such as *E. coli* and *S. aureus*. In this platform, the nanoparticle aggregation is associated with the repulsive electrostatic forces between nanoparticles, and resulted in a blue color in the presence of the pathogens (Fig. 1B)^23^,^24^. Therefore, the solution absorbs the light at longer wavelengths compared to non-aggregated form^25^. The blue and red colors are simply distinguished at a glance; thus, the presence of pathogens are detected with the naked eye, and it is further quantitatively analyzed using an image analysis tool coupled with image acquisition from a mobile phone. Thus, the current platform offers a simpler and inexpensive alternative to traditional detection methods. However, labeling with antibodies and peptides are still needed for the detection of biotargets. By employing new binders including carbohydrates and other biological recognition elements, this detection strategy will potentially be tailored to other POC applications in the resource-constrained settings.

From bacterial infection perspective, *E. coli* and *S. aureus* are the most common bacterial pathogens that pose significant clinical threats including food poisoning, skin infections and sepsis at low concentrations (10–1,000 CFUs/mL)^26^,^27^,^28^. Further, *E. coli* is reported as one of the most common species for bacterial infection observed in PD patients^29^,^30^. Here, *E. coli* spiked in PD samples were used as a clinical disease model. In end-stage renal disease (ESRD) patients, kidney replacement or dialysis to preserve any residual renal function is required. It is estimated that more than half a million patients will require dialysis in 2020 with the rising disease prevalence in the US^31^. However, a barrier in the clinical practice for broad adaption for PD has been the risk of peritonitis, *i.e.*, inflammation of the peritoneum. Peritonitis has to be detected early and treated before it becomes a chronic case. Detecting bacterial infection offers a great promise for the early diagnosis of peritonitis as well as other bacteria-induced diseases such as sepsis^26^ and ventilator-associated pneumonia^32^ at the bedside and resource-constrained settings, where time consuming and expensive assays such as cell cultures, ELISA, and nucleic acid-based tests may not be available.

### CD4^+^ T lymphocyte capture and detection on a flexible polyester film-based imaging platform

**Figure 5:**
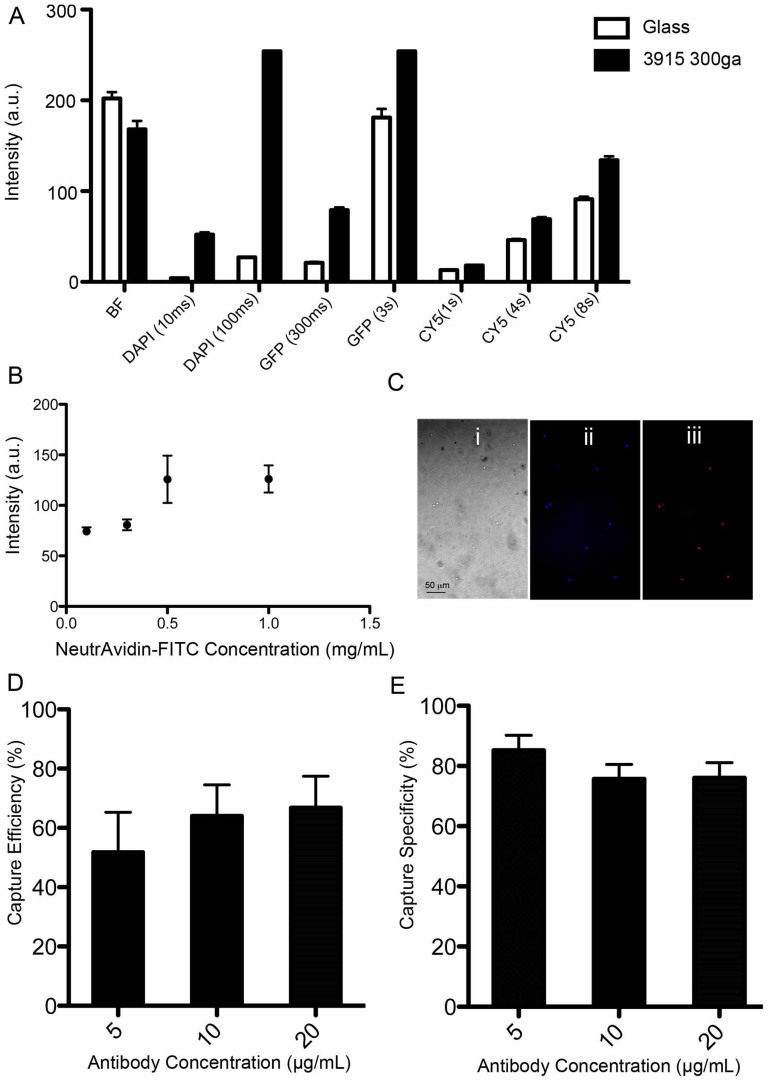
A flexible material (*i.e.*, polyester film)-based platform for the capture of CD4^+^ T lymphocytes for HIV-1 diagnosis. In the experiments, glass was determined as a standard substrate, and compared with polyester film (3915 300ga Hostaphan®). (A) Intensity plots for glass and Hostaphan®. Images are taken using various fluorescent filters (DAPI, GFP, and CY5) and exposure time. (B) Fluorescent intensity produced by surface immobilized NeutrAvidin conjugated FITC at various concentrations. (C) Fluorescent images of captured cells on these flexible film-based microfluidic devices. (i) Bright-field image, (ii) DAPI image staining nucleated cells, and (iii) Alexa647 stained CD4^+^ T lymphocytes cells. (D) Comparison of CD4^+^ T lymphocytes capture efficiency from whole blood using the platform at various anti-CD4 capture antibody concentrations. There was no statistical difference observed between the groups (n = 3, p > 0.05). (E) Comparison of capture specificity from whole blood using the platform at various anti-CD4 capture antibody concentrations. There was no statistical difference observed between the groups (n = 3, p > 0.05). Statistical assessment was performed using ANOVA with *Tukey's posthoc* test for multiple comparisons. Statistical significance threshold was set at 0.05 (p < 0.05). Error bars represent standard deviation of the mean.

A flexible polyester film with microfluidic channels was employed to selectively and efficiently capture and quantify cells of interest in unprocessed whole blood samples. Here, we evaluated this platform to capture and quantify CD4^+^ T lymphocytes in fingerprick whole blood samples for POC HIV diagnosis as a clinical disease model (Fig. 1C). We have evaluated ten different flexible substrates to fabricate microfluidic devices for capturing and counting CD4^+^ T cells from whole blood. We also evaluated these substrates for optical compatibility with wide-field of view lensless imaging on a complementary metal-oxide-semiconductor (CMOS) sensor. These flexible substrates are polyester-based transparency films that are routinely used for printing and lamination. All substrates were first imaged using bright-field (BF) microscopy to visualize any inbuilt debris (Fig. S10). Some of these substrates such as 3M, 7333 Hostaphan®, 2262N Hostaphan®, 2261N Hostaphan®, and Grafix showed debris and small inbuilt particles that could hinder detection of captured cells. As captured CD4^+^ T cells were stained with fluorescent dyes, we first evaluated these substrates for autofluorescence at various wavelengths (blue, green, and red regions) using a fluorescent microscope (Fig. S10). The substrates having large number of inbuilt debris particles also showed higher autofluorescence when samples were imaged using a fluorescent microscope (Fig. S10). To quantify the autofluorescence intensity of these images, we calculated the mean autofluorescence intensity of substrates using NIH ImageJ software (http://rsb.info.nih.gov/nih-image/). The intensity histograms, corresponding microscope images, and the mean intensity plots were demonstrated in Figures S10 and S11. Here, we used a glass slide as a standard substrate for this characterization. The substrate that showed comparable autofluorescence characteristics with glass substrates was chosen for the device assembly, and thus, 3915 300ga Hostaphan® material was used for further studies (Fig. 5A). Although 3915 Hostaphan® material resulted in higher autofluorescence at DAPI excitation, this autofluorescence was not high enough to block the strong DAPI signal. Comparing the substrate autofluorescence in green (GFP) and red (CY5) excitation, we observed that 3915 Hostaphan® material showed lesser autofluorescence in red excitation. The autofluorescence difference between glass and 3915 Hostaphan® material at red excitation was also less as compared to green excitation (Fig. 5A).

For the capture of CD4^+^ T cells, this flexible platform was assembled and functionalized to immobilize the capture antibody (Fig. 1C). To optimize the surface chemistry, we have used various concentrations of NeutrAvidin-conjugated-FITC (NA-FITC) after N-gamma-Maleimidobutyryl-oxysuccinimide ester (GMBS) functionalization step ranging from 0.1 mg/mL to 1 mg/mL. We observed an increase in fluorescent intensity up to 0.5 mg/mL NA-FITC concentration. We then selected 0.5 mg/mL NA-FITC concentration for further experiments as the fluorescent intensity saturated beyond this concentration (Fig. 5B). The assembled devices were further functionalized with various concentrations of anti-CD4 capture antibody (5, 10, and 20 μg/mL). A drop of whole blood (10 μL) was sampled into the inlet of microfluidic channels of the platform. The blood filled the whole channel due to capillary forces, without using a micro-pump (Movie S1). The flexible film-based devices were bent up and down to mix the blood inside the channels to enhance cell surface interactions to improve cell capture (Movie S2). DAPI staining was used to stain nucleated white blood cells (WBCs), whereas Alexa Flour 647 (AF647) anti-CD4 antibody was used to stain captured CD4^+^ T cells. The surfaces were imaged using a fluorescent microscope and cell capture efficiency and specificity were calculated (Supplementary Information). The bright-field and fluorescent images of the captured cells are shown in Fig. 5C. Captured WBCs and CD4^+^ T cells can be observed clearly through the flexible substrate. The CD4^+^ T cell capture efficiencies were 51.8% ± 13.5%, 64.0% ± 10.1%, and 66.7% ± 10.7% for devices functionalized with 5, 10, and 20 μg/mL capture antibody, respectively (Fig. 5D). The CD4^+^ T cell capture specificities were 85.2% ± 5.0%, 75.7% ± 4.8%, and 76.0% ± 5.1% for devices functionalized with 5, 10, and 20 μg/mL capture antibody, respectively (Fig. 5E). We observed that increasing the capture antibody concentration increased the CD4^+^ T cell capture efficiency and reduced the capture specificity. Here, this flexible material (*i.e.*, polyester film)-based platform can be integrated with CMOS lensless imaging platform for rapid detection of CD4^+^ T cells (Fig. S12A). Shadows of the captured cells are imaged by lensless sensor in a few seconds (Figs. 1C (iv) and S12B–C). The CMOS sensor allowed rapid cell detection without using a fluorescent tag. However, transparency films/substrates with higher clarity and quality would enhance captured cell shadow contrast for quantification. Overall, the developed platform allows efficient CD4^+^ T cell counting using fingerprick volume of unprocessed whole blood samples at the POC. These results indicate that the platform has the potential to replace conventional non-disposable glass-based rigid biosensing devices in the future given that the capture efficiency and specificity are further increased beyond 90% by further optimization of the materials and optical properties of the films as a substrate. In the current form, the device processing requires manual pipetting steps for blood injection and channel washing, but the developed platform can potentially be made fully automated by integrating microfluidic devices to disposable cartridges.

From HIV diagnostic perspective, accurate CD4^+^ T cell count is essential for HIV-1 diagnosis and treatment monitoring^33^,^34^,^35^. Current World Health Organization (WHO) guidelines recommend antiretroviral therapy (ART) for individuals with CD4^+^ T cell count of less than 500 cells/μl^36^. Conventional CD4^+^ T cell counting methods require flow cytometer, i.e., a gold standard method, for CD4^+^ T cell counting in the developed world. This method, however, requires a skilled operator, expensive infrastructure and reagents. The disposable and flexible biosensing platform for efficient counting of CD4^+^ T cells has potential to address some of these global health challenges at the POC settings.

## Conclusion

Paper and flexible material-based biosensing platforms are powerful tools to create affordable and disposable POC disease detection and treatment monitoring assays, particularly for the resource-constrained settings. There is a need for their integration with different detection strategies for distinct biotargets from multiple biological and clinical matrices such as whole blood, serum, saline buffer, dialysis effluent or lysates with high sensitivity and specificity. In this manuscript, as clinical models, we demonstrated the isolation and detection of HIV-1 CD4^+^ T lymphocytes, *E. coli* and *S. aureus* in various matrices including whole blood, plasma, and peritoneal dialysis fluid. We also reported various cellulose paper and flexible substrate-based platforms allowing broadly applicable diagnostic assays through seamlessly integrating optical and electrical sensing modalities. Thus, these various platforms are uniquely able to isolate and detect multiple biotargets selectively, sensitively, and repeatably from diverse biological matrices using antibodies and peptides. Further, these platforms could potentially be adapted and tailored to detect other pathogens and biotargets with well-known biomarkers^13^,^34^,^37^,^38^,^39^,^40^,^41^,^42^.

## Author Contributions

H.S., W.A., F.I., M.Y., and U.D. designed the study and the experiments; H.S., W.A., F.I., M.Y., M.S., M.J., M.H.Z. and N.G.D. performed the experiments; U.A.G. performed the SEM imaging shown in Figures S8 and S9; H.S., W.A. F.I., M.Y., and U.D. wrote the manuscript; D.R.K. supervised the virology aspect of the study and provided HIV samples; H.S., W.A., F.I., M.Y., and U.D. supervised the research.

## Supplementary Material

Supplementary Information SUPPLEMENTARY INFO
