# Supplementary material for: Paper and Flexible Substrates as Materials for Biosensing Platforms to Detect Multiple Biotargets
Source: Sci Rep. 2015 Mar 6;5:8719. doi: 10.1038/srep08719 (PMC4351531; doi:10.1038/srep08719)
Supplement: Supplementary Information — SUPPLEMENTARY INFO [file srep08719-s1.doc]

Supporting Information

**Paper and Flexible Substrates as Materials for Biosensing Platforms to Detect Multiple Biotargets**

Hadi Shafiee ^1^,†, Waseem Asghar ^2, ‡^,†, Fatih Inci ^2^,†, Mehmet Yuksekkaya ^1, ¥^, Muntasir Jahangir ^1^, Michael H. Zhang ^1^, Naside Gozde Durmus ^3,4^, Umut Atakan Gurkan ^1,#^, Daniel R. Kuritzkes ^5^, Utkan Demirci ^1,2,5^,*

^1^ Demirci Bio-Acoustic-MEMS in Medicine (BAMM) Laboratory, Division of Biomedical Engineering, Division of Renal Medicine, Department of Medicine, Brigham and Women’s Hospital, Harvard Medical School, Boston, MA, USA.

^2^ Demirci Bio-Acoustic-MEMS in Medicine (BAMM) Laboratory, Stanford University School of Medicine, Canary Center at Stanford for Cancer Early Detection, Palo Alto, California, USA.

^3^ Department of Biochemistry, Stanford School of Medicine, Stanford, California, USA.

^4^ Stanford Genome Technology Center, Stanford University, Palo Alto, California, USA.

^5^  Division of Infectious Diseases, Brigham and Women’s Hospital, Harvard Medical School, MA, USA.

†These authors contributed equally to this work.

^‡^ Present address: Asghar-Lab, Micro and Nanotechnology in Medicine, Department of Computer Engineering & Electrical Engineering and Computer Science, Florida Atlantic University, Boca Raton, FL, USA.

^¥^ Present address: Başkent University, Faculty of Engineering, Biomedical Engineering Department, Ankara, Turkey.

^#^ Present address: Case Biomanufacturing and Microfabrication Laboratory, Mechanical and Aerospace Engineering, Case Western Reserve University, Advanced Platform Technology Center, Louis Stokes Cleveland Veterans Affairs Medical Center, Cleveland, Ohio, USA.

* Corresponding author: Utkan Demirci, PhD

Demirci Bio-Acoustic-MEMS in Medicine (BAMM) Laboratory,

Stanford University School of Medicine,

Canary Center at Stanford for Cancer Early Detection,

3155 Porter Drive, Palo Alto, CA 94304, USA

Email: utkan@stanford.edu

**MATERIAL AND METHODS**

1. **Reagents**

Triton X-100 (100%), glycerol (100%), and Bovine Serum Albumin (BSA, 10%) were purchased from Sigma-Aldrich® (St Louis, MO). Dulbecco’s phosphate buffered saline (DPBS, 1X) was purchased from Life TechnologiesTM (Grand Island, NY). HyPure^TM^ Molecular Biology Grade Water was obtained from Fisher Scientific (Agawam, MA). Ethanol (200 proof) was purchased from Sigma Aldrich (Sheboygan, WI). Biotinylated polyclonal goat anti-gp120 antibody (4mg/mL) was obtained from Abcam® (ab53937, Cambridge, MA). Hostaphan® 3915, 44Rx7, 7333, 3901, 2262N and 2261N substrates were obtained from Mitsubishi Polyester (Greer, SC). Melinex® 453 substrate also was obtained from Tekra, a division of EIS, Inc. (New Berlin, WI). Grafix biodegradable matte clear acetate was purchased from Grafix (Maple Heights, OH). 3M Laser Printer Film CG5000 was purchased from 3M (St. Paul, MN). An 80 µm thick optically clear double sided adhesive (DSA, 3M 8113) was purchased from 3M (St. Paul, MN). Ethanol (200 proof) and glass slides (Gold Seal Cover glass 24 mm x 40 mm) were purchased from Fisher Scientific (Fair Lawn, NJ). (3-Mercaptopropyl) trimethoxysilane (3-MPS, CN: 175617), dimethyl sulfoxide (DMSO) and lyophilized BSA (BSA, CN:A2153) were obtained from Sigma-Aldrich® (Saint Louis, MI). N-g-Maleimidobutyryloxy succinimide ester (GMBS), and NeutrAvidin protein were obtained from Pierce Biotechnology (Rockford, IL). Dulbecco’s Phosphate buffered saline (DPBS) 1 × was purchased from Gibco (Grand Island, NY). A 49-6-diamidino-2-phenylindole (DAPI) was obtained from Invitrogen (Carlsbad, CA). Biotinylated anti-CD4 antibody (Clone 13B8.2, CN:COIM0704) was obtained from Fisher Scientific (Fair Lawn, NJ). Alexa Flour 647 (AF647) conjugated Anti-CD4 antibody (Clone RPA-T4, CN:557707) and BD FACS Lysing Solution (CN: 349202) were purchased from BD Bioscience (Becton, Dickinson and Company, San Jose, CA). 1X RBC Lysis Buffer (CN: 00-4333-57) was purchased from eBioscience (San Diego, CA). Streptavidin-coated magnetic beads (1 µm diameter) were purchased from Thermo Scientific (88816, Rockford, IL).

1. **HIV detection on a flexible polyester film-based electrical sensing platform**

***Preparing Magnetic Beads Conjugated with anti-gp120 Antibody***

Streptavidin-coated magnetic beads (1 µm diameter) were diluted with DPBS to obtain 1:10 (v/v) dilution. Then, the beads were washed three times with DPBS (1.5 mL) using a BioMag® multistep magnetic separator (Polyscience Inc., Warrington, PA). Antibody conjugation was performed by adding biotinylated polyclonal anti-gp120 antibodies with the stock concentration of 15 µg/mL to the magnetic beads solution and incubated for 2 hours at 4°C on a rotator with a speed of 30 rpm. The non-conjugated antibodies in the solution were then removed from the sample through washing with DPBS (three times).

***Capturing HIV-1 on Magnetic Beads Conjugated with anti-gp120 Antibody***

Multiple HIV-1 subtypes (A, B, C, D, E, G, and panel (cocktail of HIV-1 subtypes A, B, C, D, and circulating recombinant forms, CRF01_AE and CRF02_AG)) spiked in DPBS, whole blood, or plasma (50 µL) were mixed with conjugated magnetic beads and incubated for half an hour at room temperature on a rotator with a speed of 15 rpm. The viral load of the HIV-1 subtypes in culture media were measured as 1.74 × 10^8^ (subtype A), 1.2 × 10^8^ (subtype B), 1.17 × 10^8^ (subtype C), 2.9 × 10^8^ (subtype D), 8.39 × 10^8^ (subtype E), 6.53 × 10^8^ (subtype G), and 1.48 × 10^9^ (panel) copies/mL. To prepare the control samples, DPBS, whole blood, or plasma without viruses was mixed with conjugated magnetic beads.

To improve the detection limit in our electrical sensing microchip we increased the sample volume to 5mL to capture more viruses for detection. HIV-1 samples (subtype D, 2.9 × 10^6^ copies/mL) diluted in cultured media (4.9 mL) were mixed with magnetic beads conjugated with biotinylated polyclonal anti-gp120 antibodies (100 µL). To capture viruses, the samples were incubated for an hour at room temperature on a rotator with a rotation speed of 15 rpm. Control samples in this set of experiments were determined as streptavidin-coated magnetic beads conjugated with biotinylated polyclonal anti-gp120 antibodies in DPBS solution without virus.

***Viral Lysis and Impedance Measurement***

The mixture of HIV-1 and conjugated magnetic beads were washed four times with 20% glycerol (diluted in grade water) to remove not captured viruses in the solution and electrically conductive media. After washing step, 1% Triton-X 100 (diluted in grade water) was mixed with the conjugated magnetic beads and incubated for 5 minutes to lyse the captured viruses. The viral lysis step compromises the membrane of the virus and releases the membrane phospholipids and proteins, capsid proteins, intracellular ions, retroviral enzymes, and nucleic acids into the background solution. The viral lysate samples were then introduced into the polyester film-based platform having microfluidic channels with two rail electrodes. Impedance magnitude and phase of the signal were measured at 1 V and over a broad range of frequencies between 100 Hz and 1 MHz.

***Microchip Fabrication***

Two rail electrodes were patterned on hydrophobic transparency substrates using silver ink (CI-1001, Engineered Materials System, Delaware, OH). The masks to fabricate the electrodes were cut on a double-sided-adhesive film (DSA) using a laser cutter (Universal Laser Systems Inc., VLS2.3, Scottsdale, AZ). This mask was taped on top of a polyester film and conductive ink was poured on top of the mask to fill the openings on the DSA (**Fig. S1**). A glass cover slip was used to distribute the ink evenly everywhere in the openings. The polyester substrate with the inks were then baked in oven at 80 °C for an hour. After the ink dried, the protective DSA was removed and the electrodes patterned in the openings of the mask were left on the substrate. The width and spacing of these two rail electrodes were 2 mm and 1 mm, respectively. **Fig. S2** shows the scanning electron microscopy (SEM) of the electrodes on substrate.

The flexible polyester film-based platform with microchannels was fabricated using non-lithographic techniques. The platform has three layers; top and bottom transparency substrate layers and in between the double sided adhesive film channel layer. The inlets and outlets were cut on the transparency polyester film with a diameter of 0.6 mm and channels were cut on the DSA with 50 µm using the laser cutter. The assembled chip size was 24 mm x 40 mm.

***Electronic Circuit Model of the Flexible Polyester Film-based Electrical Sensing Platform***

An electronic circuit model was developed to simulate the electrical response of the viral lysate samples in a polyester film-based platform with microfluidic channels **(Fig. S3)**. The system’s wiring resistance, bulk resistance of the sample, and the capacitance and resistance of the double layer were considered in our model. Through this electronic circuit model, we calculated the equivalent impedance magnitude of the sample:

$$Z=\mathrm{R}_{\mathrm{e}xt}+\frac{R_{VL}}{j\omega C_{VL}}+\frac{R_{dl}\sqrt{\omega}+(1-j)\sigma}{\sqrt{\omega}[1+j\omega C_{dl}(R_{dl}+(1-j)\sigma)]}$$

where, Z is the equivalent impedance, R*_sys_* is the reader’s electrical resistance,$R_{dl}$ is the double layer resistance,$C_{dl}$ is the double layer capacitance, $\sigma$ is the electrical conductivity of the sample, $\omega$ is the frequency of the signal, and$R_{VL}$ and $C_{VL}$are the electrical resistance and capacitance of the viral lysate sample, respectively. In this model, we also considered the diffusion of the electrolytes to the surface of the electrodes on polyester film-based platforms with microfluidic channels through Warburg impedance: $Z_{W}=\frac{(1-j)\sigma}{\sqrt{\omega}}$.

In the experiments, the impedance magnitude shifts for HIV-1 subtypes A, B, C, D, E, G, and panel were measured and observed as 23 ± 3% (0.44 ± 0.06 MΩ), 32 ± 3% (0.60 ± 0.06 MΩ), 22 ± 5% (0.42 ± 0.09 MΩ), 28 ± 3% (0.53 ± 0.07 MΩ), 34 ± 6% (0.66 ± 0.12 MΩ), 28 ± 1% (0.53 ± 0.03 MΩ), 35 ± 4% (0.66 ± 0.08 MΩ), respectively (**Fig. 2D**). The viral loads of HIV-1 subtypes A, B, C, D, E, G, and panel in these experiments were 1.74 × 10^8^, 1.2 × 10^8^, 1.17 × 10^8^, 2.9 × 10^8^, 8.39 × 10^8^, 6.53 × 10^8^, and 1.49 × 10^9^ copies/mL, respectively. These results show the ability of the polyester film-based microchip to detect multiple HIV-1 subtypes spiked in DPBS at viral loads on the order of 10^8^ copies/mL.

***Repeatability Measurement***

The repeatability of the electrical sensing measurements were calculated using the following repeatability definition:


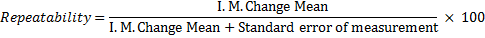


where, $I.M.$ is the impedance magnitude of the viral lysate or control samples. The repeatability measurement results are shown in **Fig. 2E**.

1. **Bacteria detection and quantification on a cellulose paper-based nanoparticle aggregation substrate and image acquisition with a mobile phone**

***Escherichia coli (E. coli) Culture***

In the experiments, we used genetically modified *E. coli*. Briefly, the *E. coli* strain BL21 Star™ was transfected using pRSET-EmGFP plasmid having ampicillin resistance and green fluorescence by incubating at 41ºC for 30 seconds. Then, the bacteria solution was transferred onto ice. The genetically modified bacteria strain was kept in LB Broth (Lennox) containing catabolite repression medium, and incubated at 37ºC in a constant shaking incubator at 250 RPM for an hour. The bacteria strain was sampled onto LB agar plate with ampicillin (100 mg/mL), and then, incubated for 16 hours at 37ºC. After bacterial grow, a single *E. coli* colony was chosen to inoculate in 5 mL of LB medium with ampicillin (100 mg/mL), and incubated in a constant shaking incubator at 250 RPM for 16 hours at 37ºC. For quantification of bacteria, the stock solution was diluted in DPBS and plated onto LB-ampicillin plates, and incubated overnight at 37ºC. The individual colonies were calculated. The stock solution was observed to be 1.5 x 10^8^ colony forming units (CFUs)/mL, and reconstituted into several concentrations ranging 8 to 1.5 x 10^6^ CFUs/mL.

***Staphylococcus aureus (S. aureus) Culture***

According to the manufacturer’s protocol, *S. aureus* (ATCC #25923, American Type Culture Collection, Mannassas, VA) cells were first hydrated, followed by their isolation on a LB agar plate. Then, a single isolated colony was chosen for the inoculation in 3 mL of LB media for 18 hours at 37⁰C, 250 rpm on an incubator shaker. Bacteria stock solution was diluted down to nine-fold in PBS for quantification. Diluted cultures were plated onto LB agar, followed by an incubation at 37°C overnight. Individual *S. aureus* colonies were then counted after overnight incubation. The concentration of stock cultures was calculated as 10^9^ CFUs/mL.

***Modification of Nanoparticles and E. coli Sampling***

100 μL of gold nanoparticles (AuNP) were first modified with 11-Mercaptoundecanoic acid (MUA) (100 μL of 1 mM MUA dissolved in ethanol), and incubated for an hour to form carboxyl groups at room temperature. For N-Ethyl-N'-(3-dimethylaminopropyl) carbodiimide hydrochloride (EDC) / N-hydroxysulfosuccinimide (NHS) (EDC/NHS) coupling reagent, 100 mM EDC and 50 mM NHS were dissolved in 50 mM MES buffer (pH 5.0). To generate succinimide groups for the binding of amine-terminated organic molecules (*e.g.*, proteins), EDC/NHS coupling reagent was added to MUA activated AuNP solution, and this mixture was incubated for an hour at room temperature. To generate the recognition between the modified AuNPs and *E. coli*, 40 μL of lipopolysaccharide binding protein (LBP) (10 μg/mL) was added to the solution. Then, 100 μL of *E. coli* samples ranging from 8 to 1.5 x 10^6^ colony forming units (CFUs)/mL were added to the modified nanoparticle solution.

***Quantitative Mobile Phone-based Image Analysis on Cellulose Paper-based Platform***

To image the nanoparticle aggregation spots, a commercial cell phone (Sony Ericson i790) was utilized. The nanoparticle solutions including *E. coli*-spiked and control (*E. coli*-free) solutions were first sampled on a cellulose paper, and the droplets were incubated for 10 minutes to be dried at room temperature. Then, the paper was placed in a black box, and brightness/illumination was adjusted by LED light. The color intensities of red, green, and blue pixel values were calculated using a customized MATLAB (MathWorks, Natick, MA) code. This code reports red, green, and blue pixel values of each spot within seconds, and presents as mean value ± standard deviation. Here, we utilized from the red (R) pixel intensity value in the data analysis, since this color value demonstrated the broadest range of color intensity for the detection experiments.

***Spectral Measurements and Data Analysis***

Spectral measurements for MUA, EDC/NHS and LBP steps were performed using Varioskan® Flash Spectral Scanning Multimode Readers, Thermo Scientific. The measuring mode was adjusted to scan the extinction changes per wavelength from 400 nm to 700 nm. Each binding event was determined with a detectable shift at the maximum extinction point of gold nanoparticles, and the results were presented as mean value ± standard error of the mean in the plot.

***Sample preparation for Scanning Electron Microscopy (SEM) imaging***

After *E. coli* was treated with the modified gold nanoparticle solution, the mixture was sampled onto thin glass cover slides. The sample was dried under a laminar flow hood for 30 minutes, and then, mounted on aluminum SEM stubs (Ted Pella Inc., Redding, CA) with carbon tape. Before SEM imaging, these samples were coated using sputter instrument (Cressington Scientific Instruments Ltd., Watford, England) with Platinum/Palladium at 40 mA for 90 seconds under Argon gas atmosphere. Then, the samples were visualized using field emission SEM (Ultra 55, Carl Zeiss MicroImaging, LLC, Thornwood, NY).

1. **CD4+ T lymphocyte capture and detection on a flexible polyester film-based imaging platform**

***Surface Chemistry to Capture CD4^+^ T lymphocytes***

The bottom transparency polyester film substrate was cleaned with ethanol and distilled water, respectively, and then, followed by drying under nitrogen gas. To form the hydroxyl (-OH) surface functional groups, the substrate surface was first treated with oxygen plasma (100 mW, 1% oxygen) for 2 minutes in a PX-250 chamber (March instruments, Concord, MA). Then, it was incubated for 30 minutes at room temperature with silanization solution (4% (v/v) 3-MPS in ethanol) in a petri dish for covalent binding. After silanization, the substrate was washed with 1 mL of ethanol. Transparency substrates were then assembled using DSA to form microfluidic channels as shown in **Fig. 1C**. Glass-based platforms were assembled and functionalized similarly to the substrate-based platforms.

Following chip assembly, the microfluidic channels were washed three times with DPBS. GMBS solution (4% (v/v) GMBS dissolved in 10% DMSO in DPBS) was injected into channels, and devices were incubated for 30 minutes at room temperature, followed by washing three times with DPBS. NeutrAvidin solution (0.1 mg/mL in DPBS) was injected into the channels and devices were incubated for 2 hours at 4°C, followed by three times DPBS wash. As a CD4^+^ T cell capturing agent, biotinylated anti-CD4 antibody (10 µg/mL in DPBS) was pipetted into the microchannels and incubated at room temperature for 30 minutes. To reduce nonspecific binding, devices were incubated with 1% (v/v) BSA in DPBS for 30 minutes at room temperature. The microchannels were washed again three times with DPBS buffer, and after this final wash, the flexible substrate-based platform was ready for cell capture experiments.

***Autofluorescence and Intensity Analysis***

To determine the autofluorescence and clearness of the substrates, microscope images are taken in different settings in terms of various filters and exposure time (*i.e.,* bright-field, UV (359 nm/461 nm) for 10ms and 100 ms, GFP (460 nm/480 nm) for 300 ms and 3s, CY5(639 nm/650 nm) for 1 s, 4 s and 8 s). The mean and standard deviation values of intensities were obtained using ImageJ software (National Institute of Health, <http://rsbweb.nih.gov/ij/>).

***CD4^+^ T Lymphocyte Capture, Imaging and Counting***

Fresh collected whole blood was pipetted into the channels and incubated for 25 minutes at room temperature. Channels were then washed three times to remove any unattached cell. Cells were fixed with 10% (v/v) BD FACS lysing solution in DPBS for 10 minutes. After fixing step, cells were stained with 0.2% (v/v) DAPI solution (5 mg DAPI stock in 1 mL of deionized water) and 1% (v/v) AF647 conjugated anti-CD4 (100 µg/mL AF647 in DPBS) for 90 minutes at 4°C. After each incubation step, microfluidic channels were washed three times with DPBS.

Fluorescent microscope (Carl Zeiss microscope, Jena, Germany) was used for bright-field and fluorescent imaging. DAPI and AF647 images of captured cells were taken with UV (359 nm/461 nm) and CY5 (639 nm/650 nm) filters. DAPI (blue) stained cells indicated the captured white blood cells (WBC, nucleated cells), whereas AF647 (red) stained cells indicated the captured CD4^+^ T cells. After manual counting of cells, the ratio of CD4^+^ T cells to WBC (Red/Blue) provided the capture specificity. Capture efficiency was calculated by dividing captured CD4^+^ T cells to initial number of injected CD4^+^ T cells multiplied by 100.

To determine initial CD4^+^ T cell count in whole blood, WBCs were isolated by incubating whole blood with 10% RBC lysis solution for 3 minutes. The cell suspension was centrifuged at 150 g for 5 minutes. Supernatant was removed and the cell pellet was re-suspended with same volume of DPBS as initial blood volume. The isolated WBCs were fixed 10% BD FACS lysing solution for 5 minutes. Cells were stained with 0.2% (v/v) DAPI solution (5 mg DAPI stock in 1 mL of deionized water) and 1% (v/v) 100 µg/ml AF647-anti-CD4 in DPBS solution for 90 min at 4°C. Between each step washing was performed by centrifuging the sample at 150 g for 5 minutes following by discarding supernatant and re-suspending the pellet. Finally, DAPI stained WBC and AF647 stained CD4^+^ T cells were counted using hemocytometer under fluorescent microscope.

***Integration of Lensless Imaging to Flexible Polyester Film-based Platform***

Lensless CMOS sensor (IDS imaging development systems, Germany, UI-1492LE-M) was integrated with the polyester substrate-based platform for rapid cell counting (**Fig. S12A**). The sensor has wide field-of-view and detects the shadows of captured cells over an area of 6.4 mm x 4.6 mm at a time with pixel size = 1.67 µm. A 3D printer (Replicator 2, Makerbot, Boston, MA) was used to build a stage to hold the CMOS sensor and a microfluidic chip in place. The light emitted by an LED (RadioShack) passes through the top transparent substrate and reaches the cells captured on bottom transparent. The shadows of the captured cells are produced and acquired by CMOS sensor.

1. **Statistical Analysis**

For the evaluation of the statistical difference between the comparison groups, we analyzed the experimental results (n = 3-10) using analysis of variance (ANOVA) with *Tukey’s posthoc* test and Mann Whitney t test for multiple comparisons. Further, in bacteria experiments, ANOVA with *Tukey*’s *posthoc* test was followed by Bonferroni's multiple comparison test for equal variances for multiple comparisons. Statistical significance threshold was set at 0.05 (p<0.05).


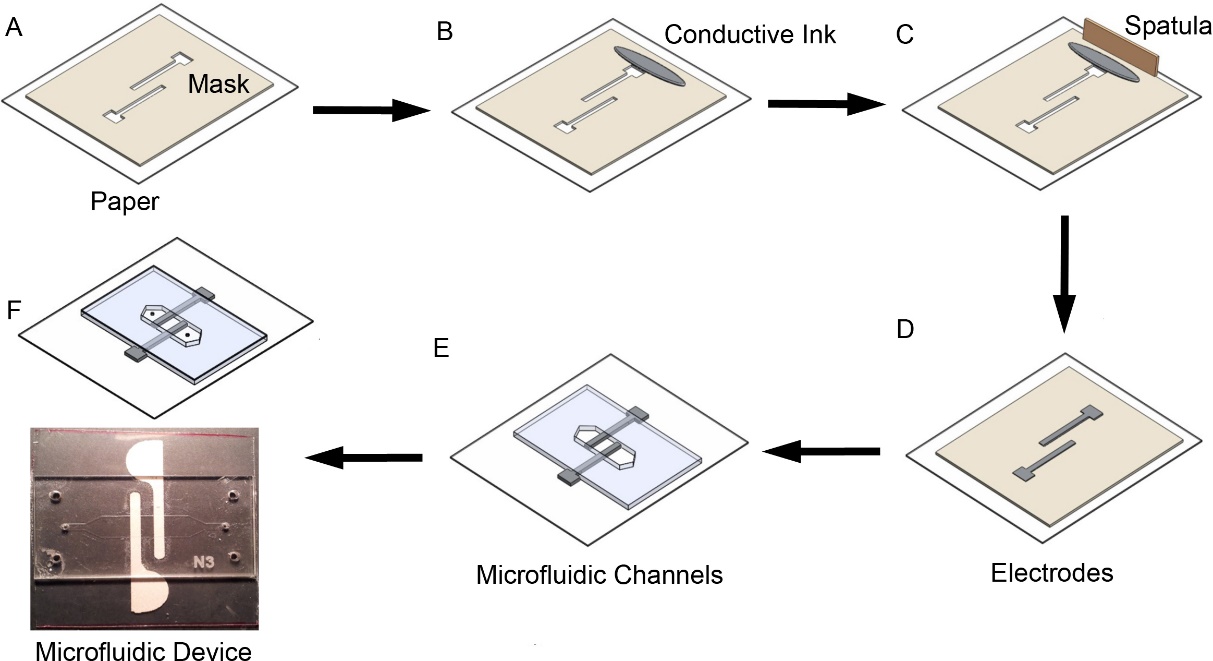


**Fig. S1.** 3D schematic of fabrication of flexible polyester film-based platform with flexible electrodes in microfluidic channels. Two rail electrodes were patterned on hydrophobic transparency substrates using silver ink. **(A)** The electrodes geometry and design were cut on a double-sided-adhesive film (DSA) using a laser cutter. **(B)** This DSA used as a mask and taped on top of a polyester substrate and conductive ink (silver) was poured on top of the mask to fill the openings on the DSA. **(C)** A glass cover slip was used to distribute the ink evenly everywhere in the openings. **(D)** The substrate with the inks were then baked in oven at 80 °C for an hour. **(E)** After the ink dried, the protective DSA was removed and the electrodes patterned in the openings of the mask were left on the substrate. Microfluidic channel was cut on a DSA layer and attached to the substrate and on top of the electrodes. Inlet and outlet to access to the channel were cut on a polyester substrate and attached to the DSA to create a closed microchannel. The thickness of the electrodes was approximately 50 µm. The width and spacing of these two rail electrodes were 2 mm and 1 mm, respectively.


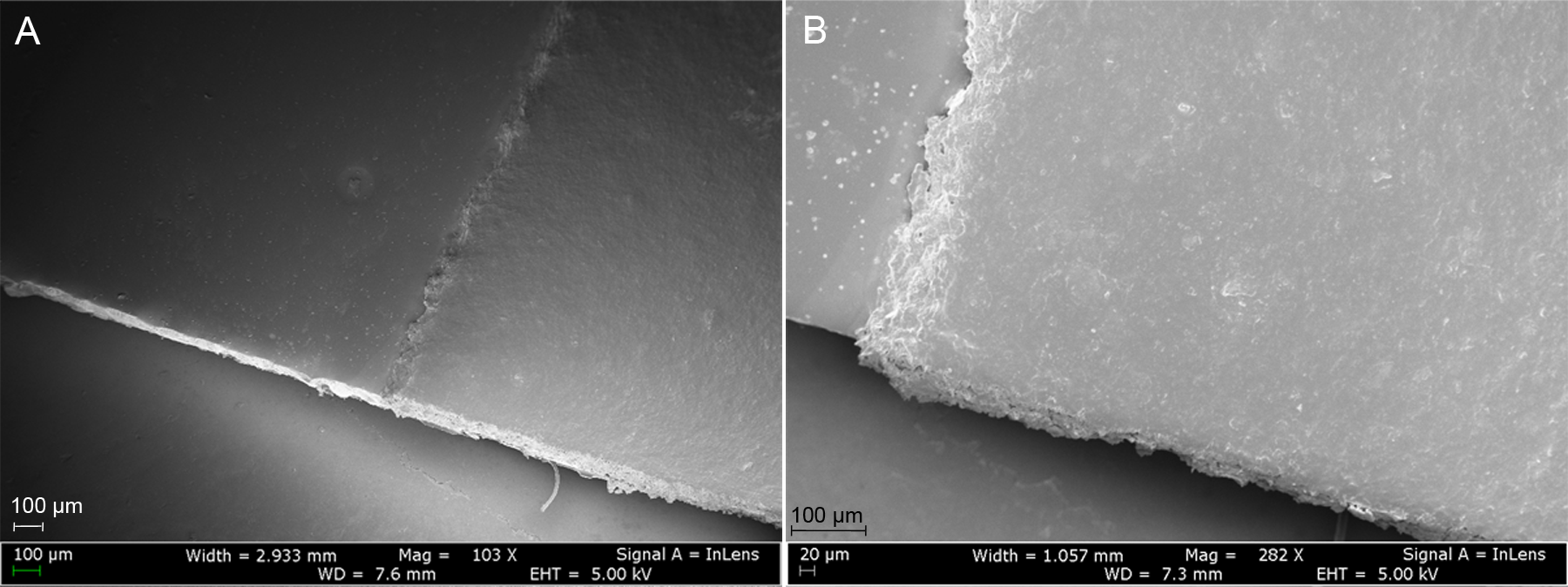


**Fig. S2.** SEM images of silver electrodes on flexible polyester substrate-based microfluidic devices. SEM images were taken at 5 KV accelerating voltage and 7.6 mm **(A)**, 7.3 mm **(B)** working distance. Magnification was 103 X **(A)** and 282 X **(B)** and signal was InLens.

**
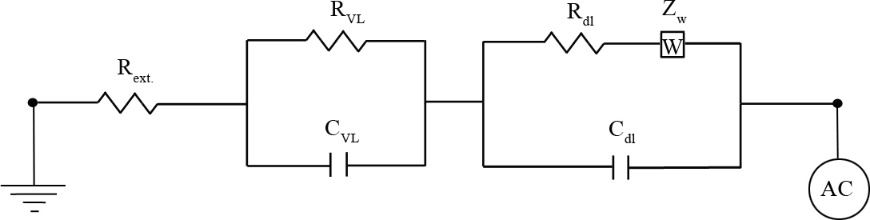
**

**Fig. S3.** Electronic circuit model of the flexible polyester film-based platform using electrical sensing approach.


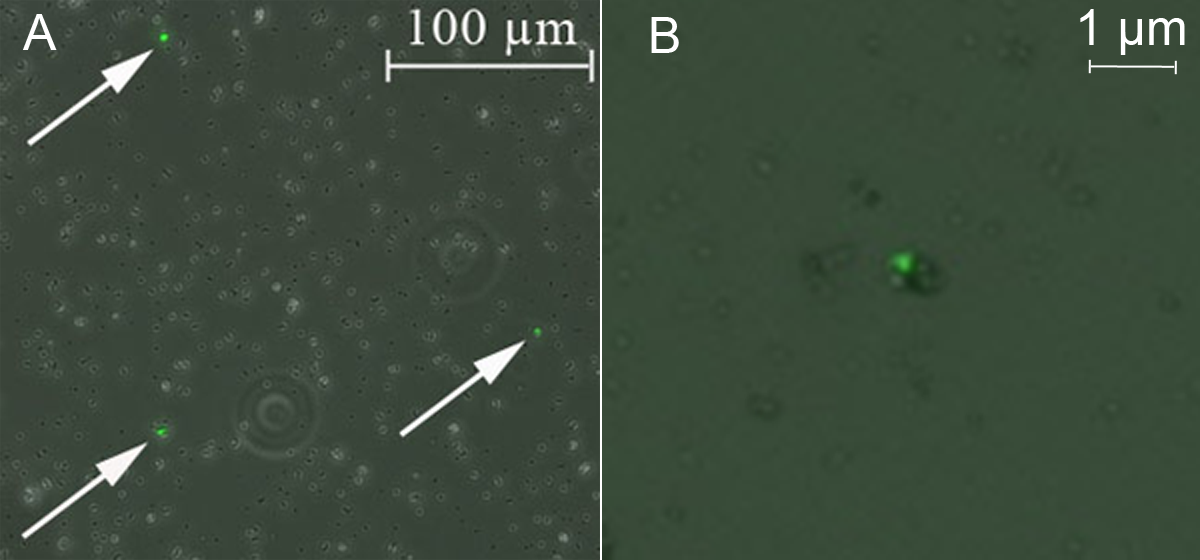


**Fig. S4.** Fluorescent images of GFP-tagged HIV-1 captured on the surface of the streptavidin-coated magnetic beads conjugated with biotinylated polyclonal anti-gp120 antibody taken using 10x **(A)** and 100x **(B)** objectives. Arrows show GFP-tagged captured HIV-1 on magnetic beads conjugated with antibodies.

**
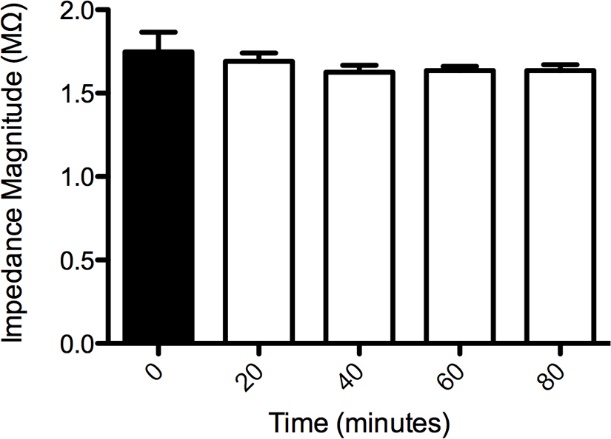
**

**Fig. S5.** Effect of time on the impedance magnitude of Triton X-100. Impedance magnitude of 1% Triton X-100 samples were measured at 1,000 Hz and 1 V every 20 minutes for 80 minutes. Statistical assessment on the results was performed using ANOVA with *Tukey’s posthoc* test for multiple comparisons. Statistical significance threshold was set at 0.05, p<0.05. There was no significant difference between the impedance magnitudes of the samples over the 80 minutes period (n=3, p>0.05).


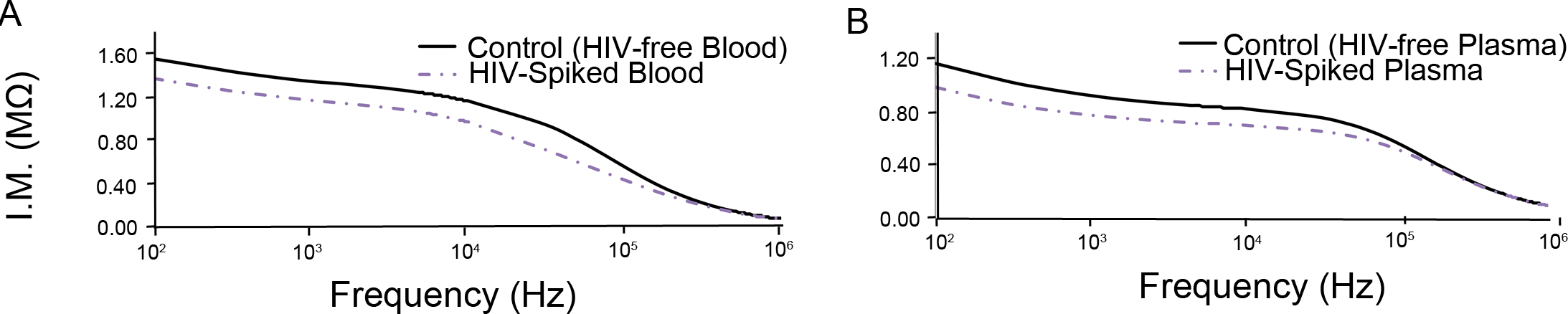


**Fig. S6.** Impedance spectroscopy for HIV spiked into whole blood and plasma samples. Average impedance magnitude spectrum of control samples and lysed HIV-1 subtype C (1.17 × 10^8^) spiked in blood **(A)** and plasma **(B)** for frequencies between 100 and 1 MHz. Control samples were HIV-free blood and HIV-free plasma for experimental results shown in **(A)** and **(B)**, respectively.

**
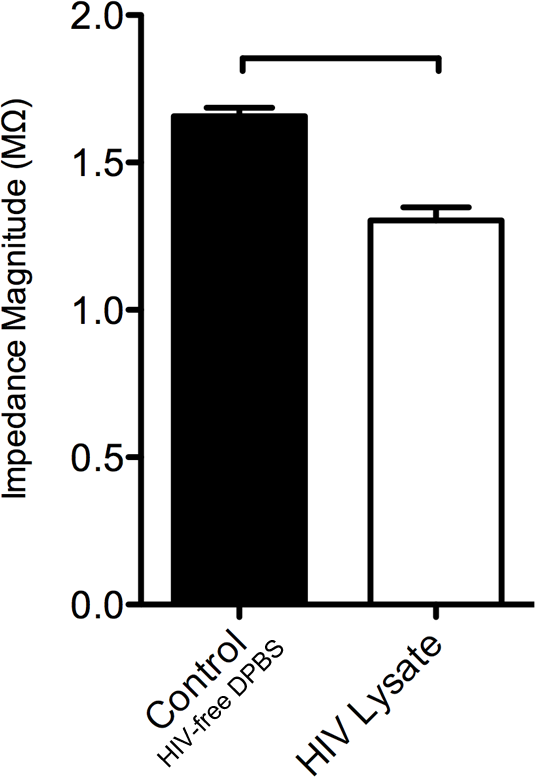
**

**Fig. S7.** Average impedance magnitude of the HIV-1 lysate (subtype D) with viral load of 2.9 × 10^6^ copies/mL at 1,000 Hz and 1 V. The sample volume was increased to 5 mL for this virus concentration to generate a detectable impedance magnitude change compared to control samples. Control samples were HIV-free DPBS. Error bars represent standard deviation of the mean (n=3). Brackets connecting individual groups indicate statistically significant impedance magnitude. Statistical significance threshold was set at 0.05, p<0.05.


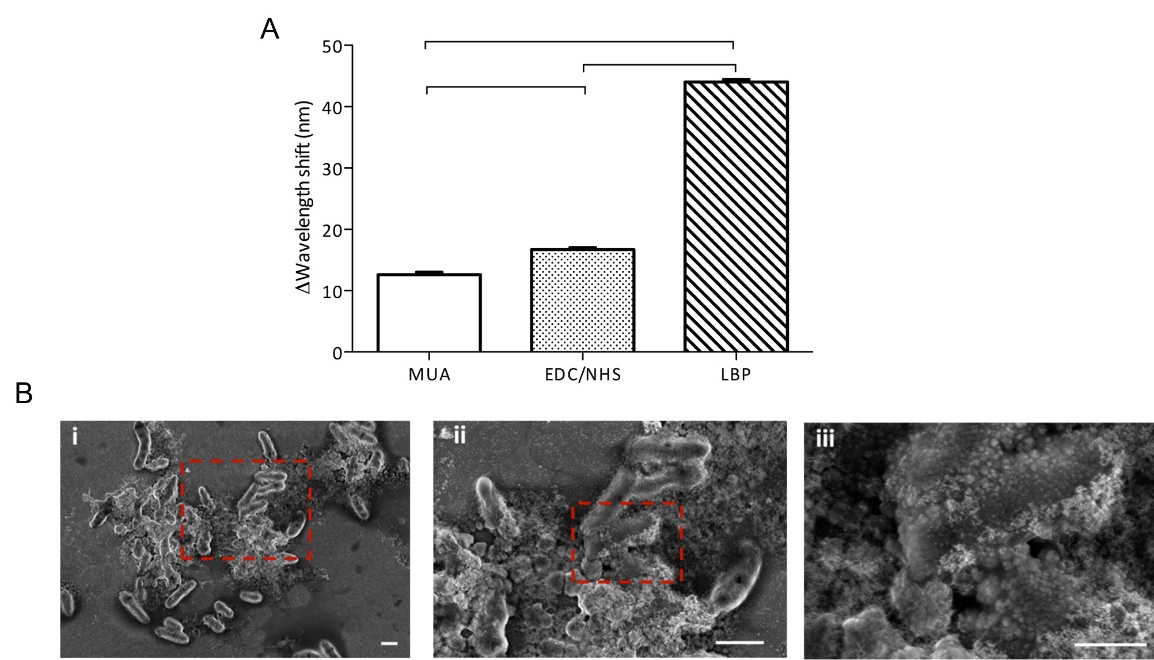


**Fig. S8.** Generation of nanoparticle aggregation concept for the paper-based detection and layer-by-layer surface modification. **(A)** Gold nanoparticles were modified with 11-Mercaptoundecanoic acid (MUA), N-Ethyl-N'-(3-dimethylaminopropyl) carbodiimide hydrochloride (EDC), and N-hydroxysulfosuccinimide (NHS), and lipopolysaccharide binding protein (LBP), respectively. Corresponding wavelength shifts were calculated and presented in the plot. After 11-Mercaptoundeconoic acid (MUA) modification, 12.6 ± 0.4 nm wavelength shift was observed compared to the extinction intensity peak value of gold nanoparticles (520 nm). Second chemical modification step (*i.e.,* EDC/NHS) coupling) resulted in 16.7 ± 0.3 nm wavelength shift, which represented a statistically significant difference (n=10, p<0.05) from the MUA step. LBP, which binds onto lipopolysaccharide residues of *E. coli* surface, was used as recognition element. At the end of LBP modification, a statistically significant wavelength peak shift was observed as 44 ± 0.4 nm with respect to the EDC/NHS step (n=10, p<0.05). Statistical assessment on the results was performed using ANOVA with Tukey’s posthoc test, followed by Bonferroni's multiple comparison test for equal variances for multiple comparisons. Statistical significance threshold was set at 0.05, p<0.05. Brackets connecting individual groups indicate statistically significant peak shift. Error bars represent standard error of the mean. **(B)** SEM imaging of the modified gold nanoparticles and *E. coli* detection on the platform. **(i)** Addition of *E. coli* caused the aggregation of gold nanoparticles. Scale bar represents 1µm. **(ii)** and **(iii)** Higher magnifications of the dashed regions were presented. SEM images were taken at 4.4 - 5 mm working distance and 3.00 – 5.00 kV accelerating voltage. Scale bars represent 1 and 0.5 µm, respectively.

**
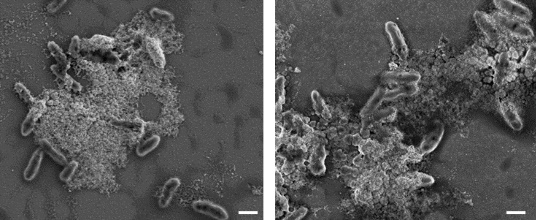
**

**Fig. S9.** Additional SEM images of modified gold nanoparticles and *E. coli* detection on the platform. SEM images were taken at 5 mm working distance and 3.00 kV accelerating voltage. Scale bars represent 1 µm.

|  | 1. **Glass Slide Images** | **Histogram** | 1. **3M Film Images** | **Histogram** |
| --- | --- | --- | --- | --- |
| **Bright Field** | 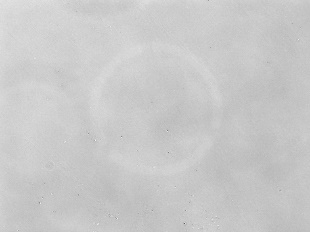 | 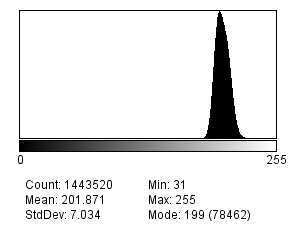 | 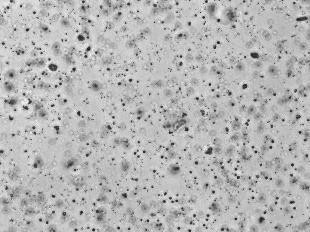 | 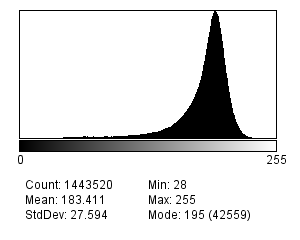 |
| **DAPI filter10ms** | 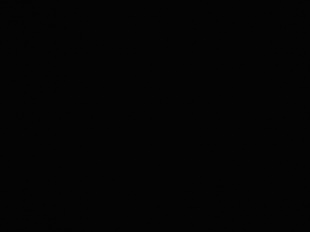 | 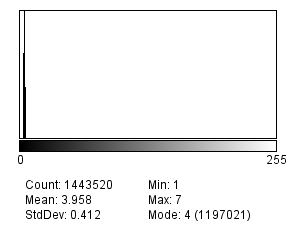 | 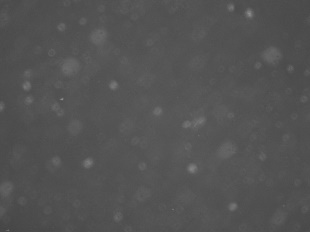 | 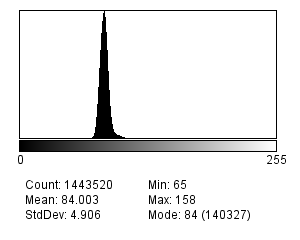 |
| **DAPI filter 100ms** | 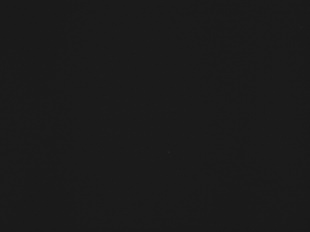 | 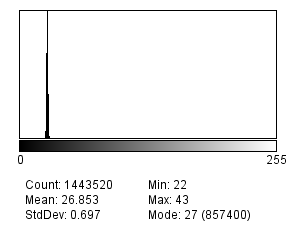 | 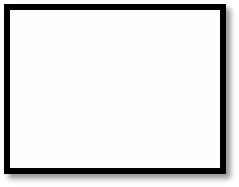 | 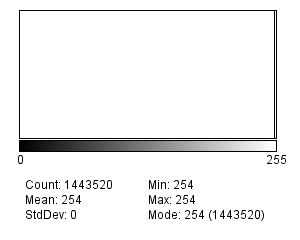 |
| **GFP filter 300ms** | 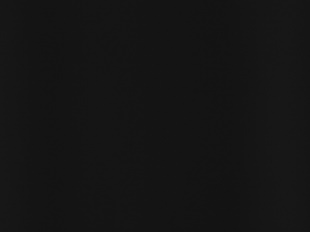 | 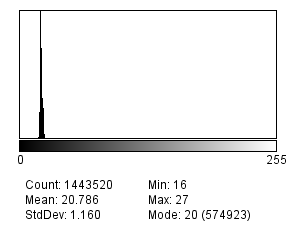 | 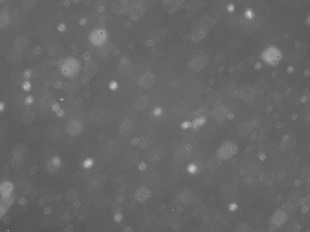 | 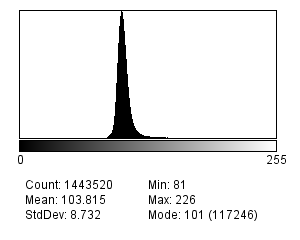 |
| **GFP filter 3s** | 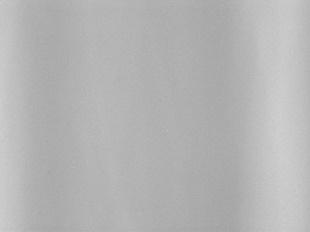 | 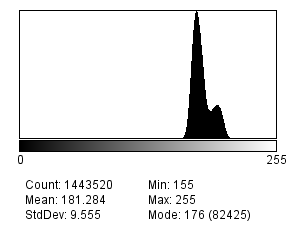 | 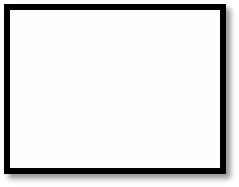 | 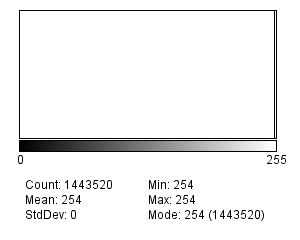 |
| **CY5 filter 1s** | 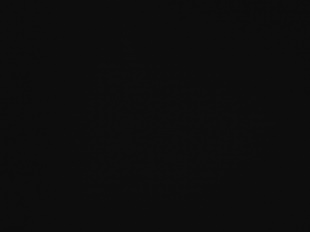 | 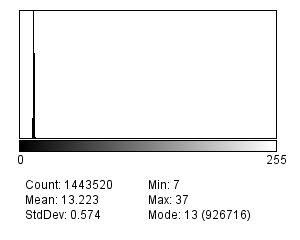 | 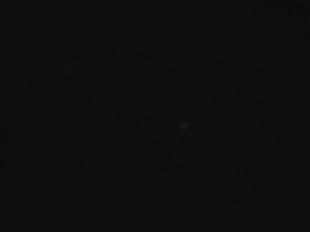 | 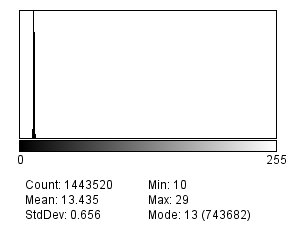 |
| **CY5 filter 4s** | 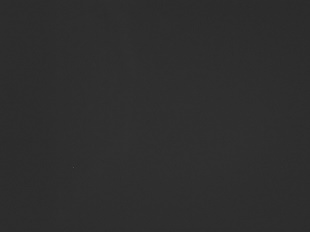 | 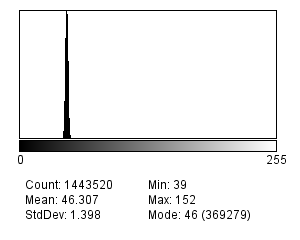 | 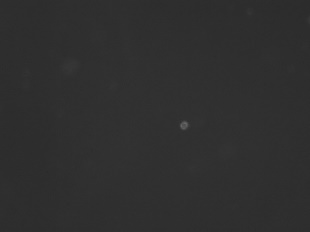 | 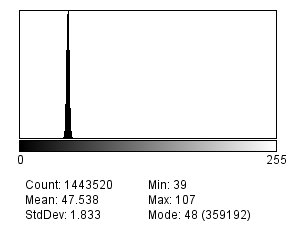 |
| **CY5 filter 8s** | 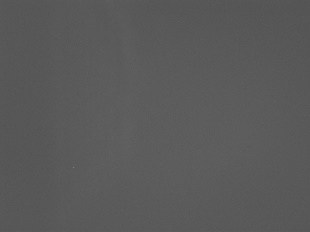 | 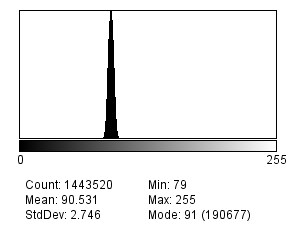 | 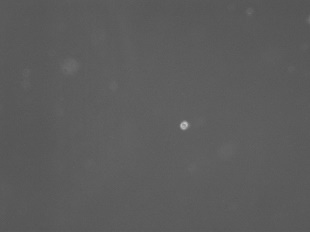 | 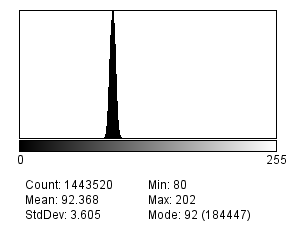 |
|  | 1. **44Rx7 Film Images Hostaphan®** | **Histogram** | 1. **7333 Film Images Hostaphan®** | **Histogram** |
| **Bright Field** | 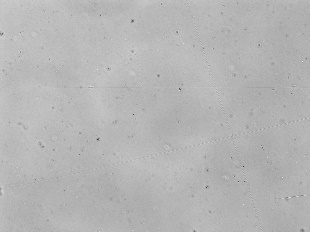 | 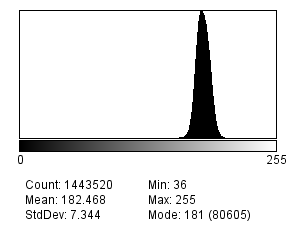 | 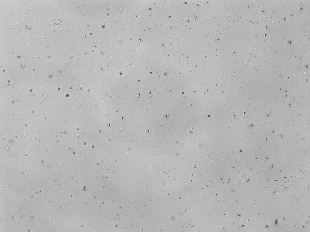 | 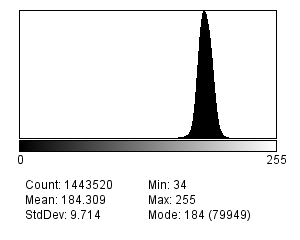 |
| **DAPI filter10ms** | 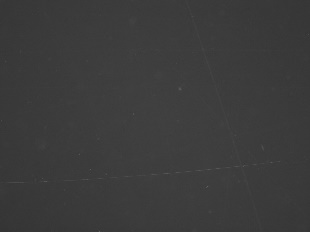 | 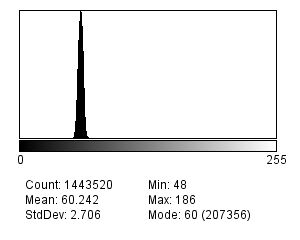 | 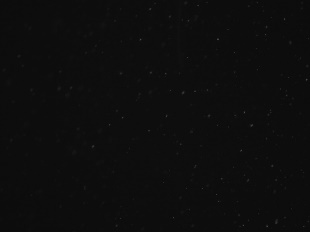 | 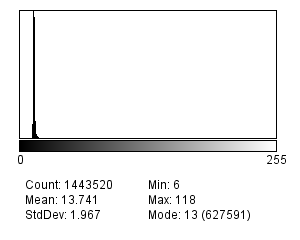 |
| **DAPI filter 100ms** | 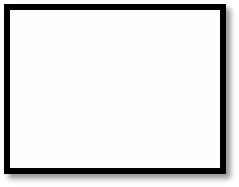 | 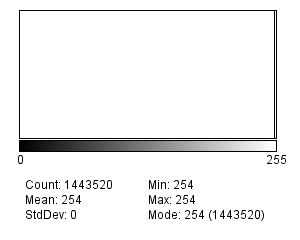 | 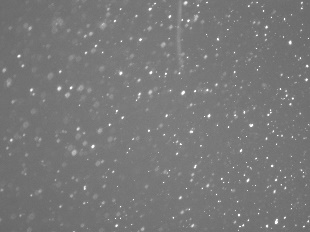 | 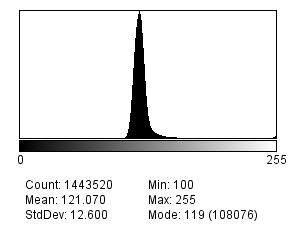 |
| **GFP filter 300ms** | 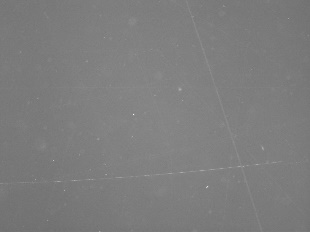 | 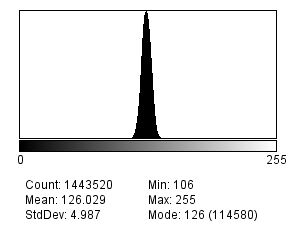 | 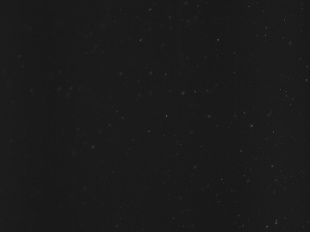 | 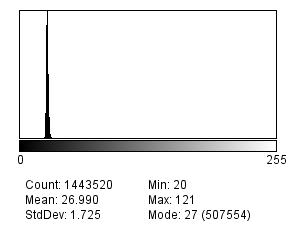 |
| **GFP filter 3s** | 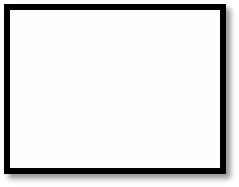 | 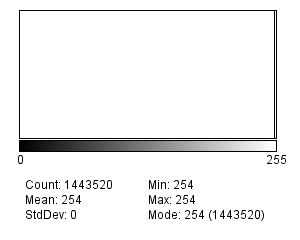 | 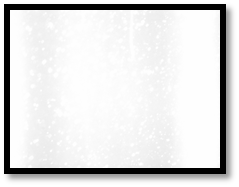 | 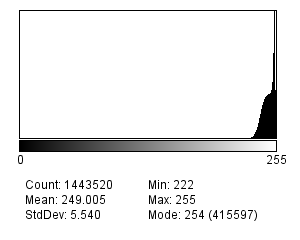 |
| **CY5 filter 1s** | 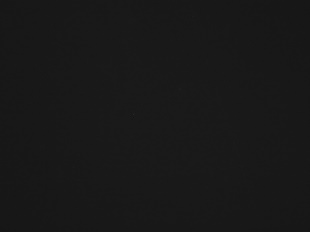 | 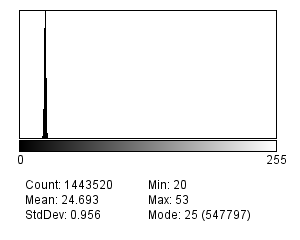 | 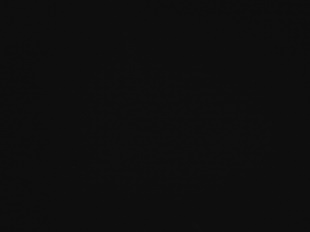 | 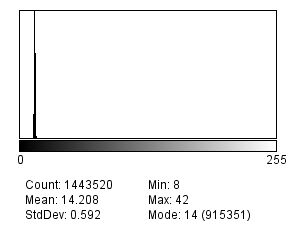 |
| **CY5 filter 4s** | 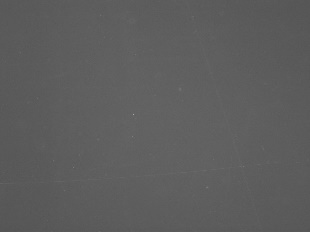 | 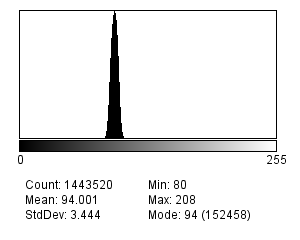 | 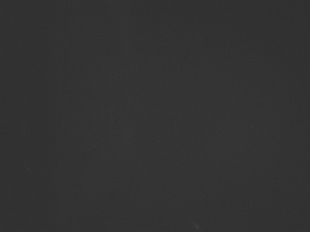 | 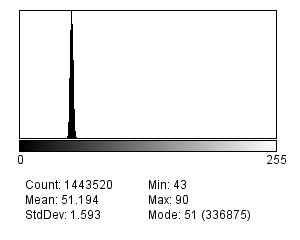 |
| **CY5 filter 8s** | 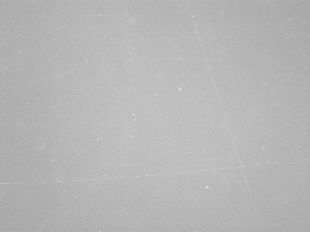 | 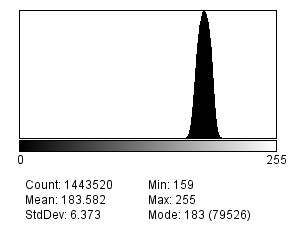 | 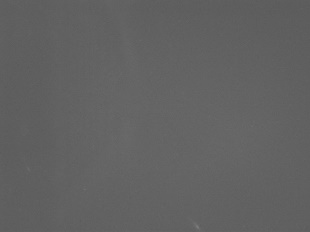 | 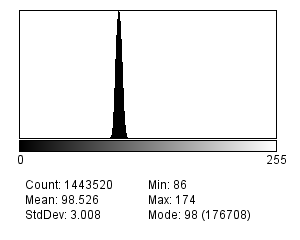 |
|  | 1. **3901 Images**   **Hostaphan®** | **Histogram** | 1. **2262N Images Hostaphan®** | **Histogram** |
| **Bright Field** | 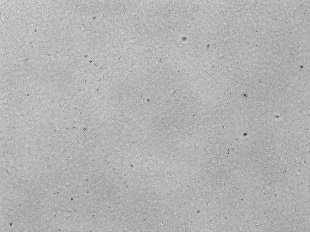 | 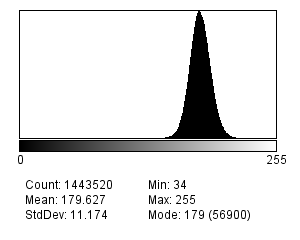 | 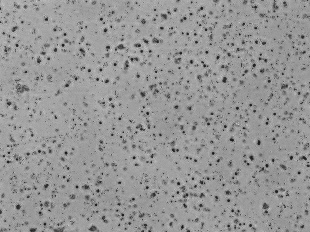 | 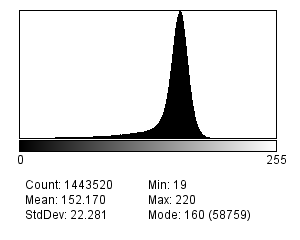 |
| **DAPI filter10ms** | 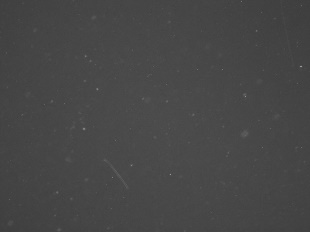 | 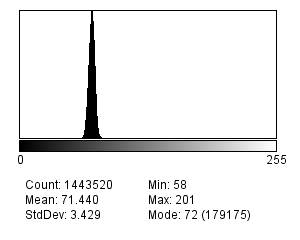 | 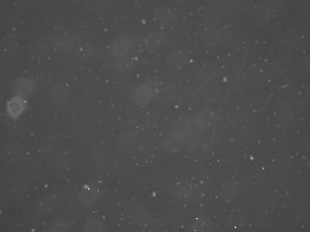 | 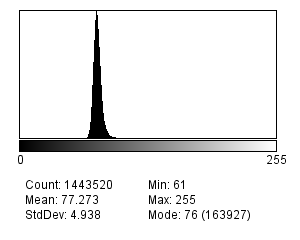 |
| **DAPI filter 100ms** | 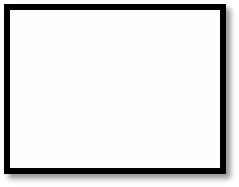 | 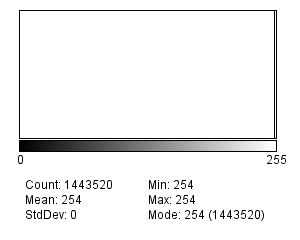 | 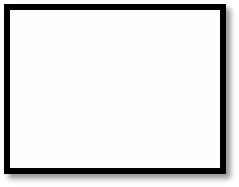 | 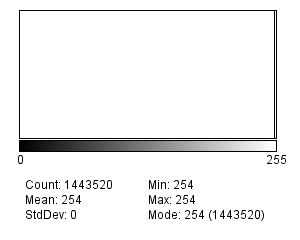 |
| **GFP filter 300ms** | 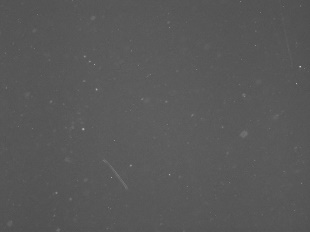 | 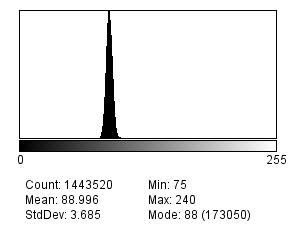 | 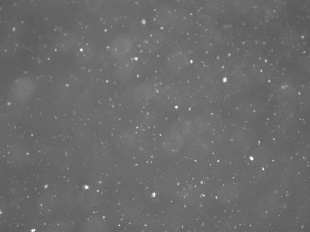 | 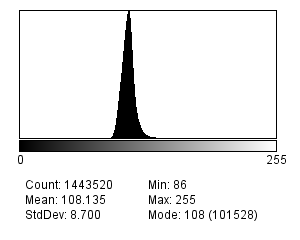 |
| **GFP filter 3s** | 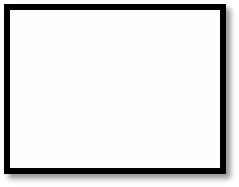 | 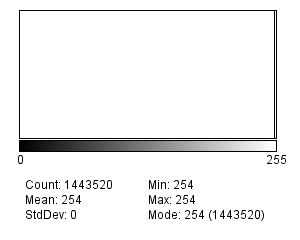 | 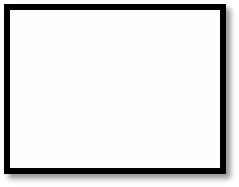 | 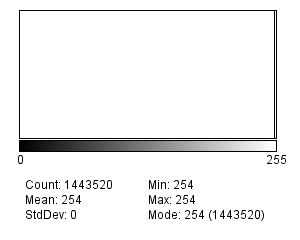 |
| **CY5 filter 1s** | 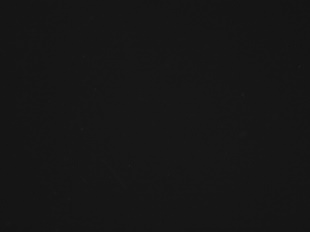 | 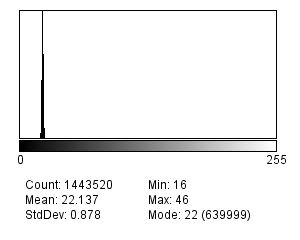 | 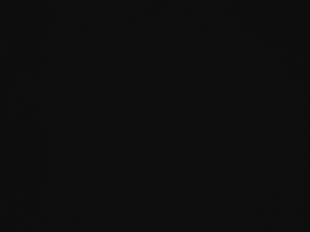 | 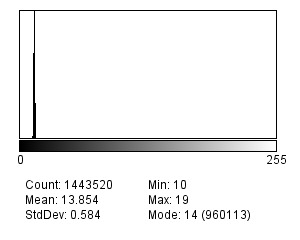 |
| **CY5 filter 4s** | 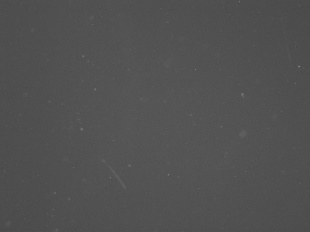 | 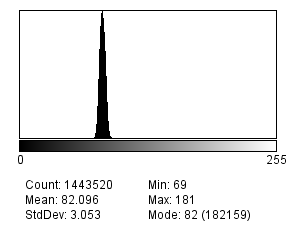 |  |  |
| **CY5 filter 8s** |  |  |  |  |

|  | 1. **3915 200ga Images Hostaphan®** | **Histogram** | 1. **3915 300ga Images Hostaphan®** | **Histogram** |
| --- | --- | --- | --- | --- |
| **Bright Field** |  |  |  |  |
| **DAPI filter10ms** |  |  |  |  |
| **DAPI filter 100ms** |  |  |  |  |
| **GFP filter 300ms** |  |  |  |  |
| **GFP filter 3s** |  |  |  |  |
| **CY5 filter 1s** |  |  |  |  |
| **CY5 filter 4s** |  |  |  |  |
| **CY5 filter 8s** |  |  |  |  |
|  | 1. **2261N Images Hostaphan®** | **Histogram** | 1. **Tekra Images Melinex® 453** | **Histogram** |
| **Bright Field** |  |  |  |  |
| **DAPI filter10ms** |  |  |  |  |
| **DAPI filter 100ms** |  |  |  |  |
| **GFP filter 300ms** |  |  |  |  |
| **GFP filter 3s** |  |  |  |  |
| **CY5 filter 1s** |  |  |  |  |
| **CY5 filter 4s** |  |  |  |  |
| **CY5 filter 8s** |  |  |  |  |

|  | 1. **Grafix** | **Histogram** |
| --- | --- | --- |
| **Bright Field** |  |  |
| **DAPI filter10ms** |  |  |
| **DAPI filter 100ms** |  |  |
| **GFP filter 300ms** |  |  |
| **GFP filter 3s** |  |  |
| **CY5 filter 1s** |  |  |
| **CY5 filter 4s** |  |  |
| **CY5 filter 8s** |  |  |

**Fig. S10**. Optical analysis of 11 different polyester based transparency flexible polyester substrates using bright-field, DAPI, GFP, and CY5 filters. The incident light intensity was varied from 10 ms to 8 s for **(A)** Glass slide, **(B)** 3M film, **(C)** 44Rx7 Hostaphan® film, **(D)** 7333 Hostaphan® film, **(E)** 3901 Hostaphan® film, **(F)** 2262N Hostaphan® film, **(G)** 3915 200ga Hostaphan® film, **(H)** 3915 300ga Hostaphan® film, **(I)** 2261N Hostaphan® film, **(J)** Melinex® 453 Tekra film, and **(K)** Grafix film.

**Fig. S11.** Autofluorescence intensities of different transparency polyester films. The presented film substrates were 3M film, 44Rx7 Hostaphan® film, 7333 Hostaphan® film, 3901 Hostaphan® film, 2262N Hostaphan® film, 3915 200ga Hostaphan® film, 3915 300ga Hostaphan® film, 2261N Hostaphan® film, Melinex® 453 Tekra film, and Grafix film and glass slide. The intensities were calculated by taking images using various fluorescent microscope filters (DAPI, GFP, and CY5) and exposure time.

**Fig. S12.** Integration of lensless imaging technology into the flexible polyester film-based platform. **(A)** CMOS lensless imaging setup attached with portable laptop. **(B)** Fluorescent image of DAPI stained WBCs inside polyester film-based platform with microfluidic channels (arrows). **(C)** CMOS shadow image of capture WBCs inside the channels (arrows).

**Table S1.** Representative material cost for the flexible polyester film-based platform using electrical sensing system.

| **Material** | **Amount used per test** | **Cost (cents)** |
| --- | --- | --- |
| Streptavidin-coated magnetic beads | 5 µL | 80 |
| Anti gp-120 antibody | 0.25 µL | 87 |
| Silver electrodes | 0.05 grams | 6 |
| Polyester Film | 2 cm × 1 cm | 0.2 |
| DSA | 2 cm × 1 cm | 0.6 |
| **Total** |  | 173.8 |
